# Supplementary figures and images for: The genomic landscape at a late stage of stickleback speciation: High genomic divergence interspersed by small localized regions of introgression
Source: PLoS Genet. 2018 May 23;14(5):e1007358. doi: 10.1371/journal.pgen.1007358 (PMC5988309; doi:10.1371/journal.pgen.1007358)

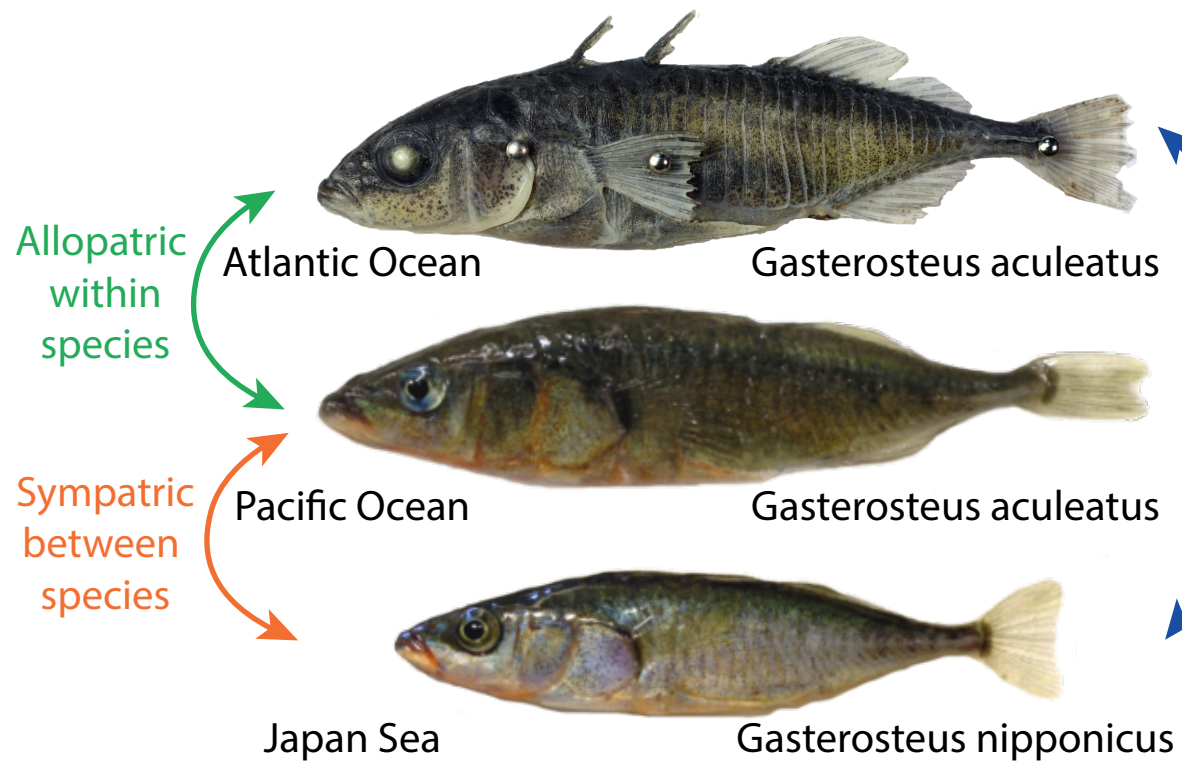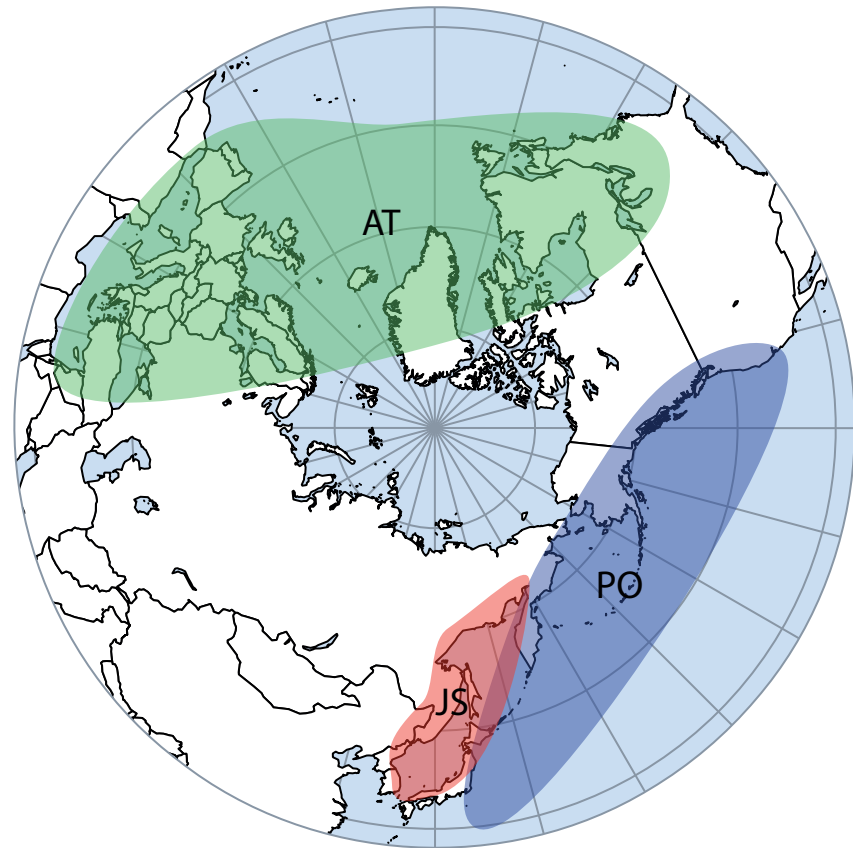

Supplement: S1 Fig — The global distribution of the Pacific and Atlantic Ocean lineages of G. aculeatus allow sympatric and allopatric comparisons with G. nipponicus; AT = Atlantic Ocean, PO = Pacific Ocean and JS = Japan Sea. (PDF) [file pgen.1007358.s008.pdf]

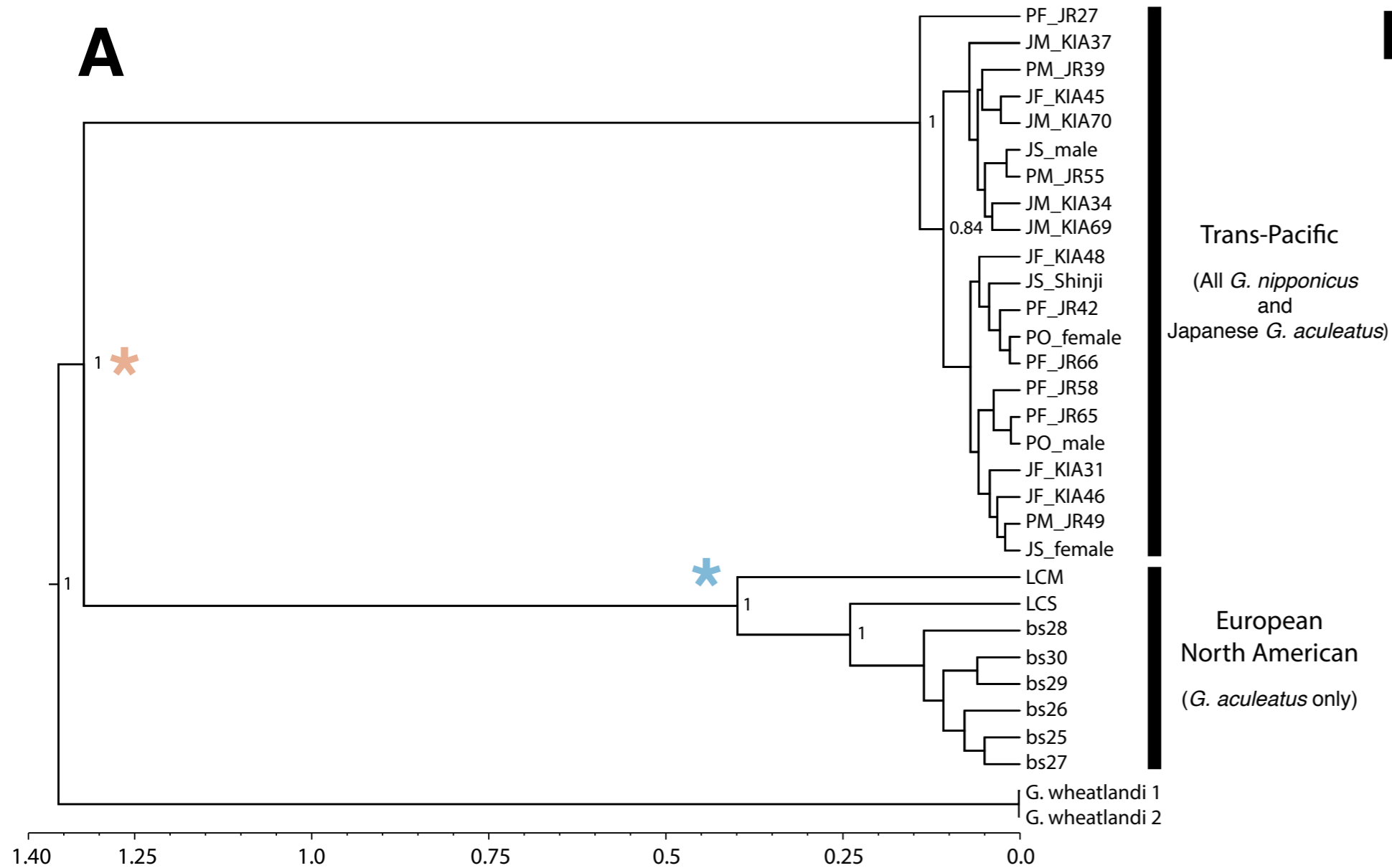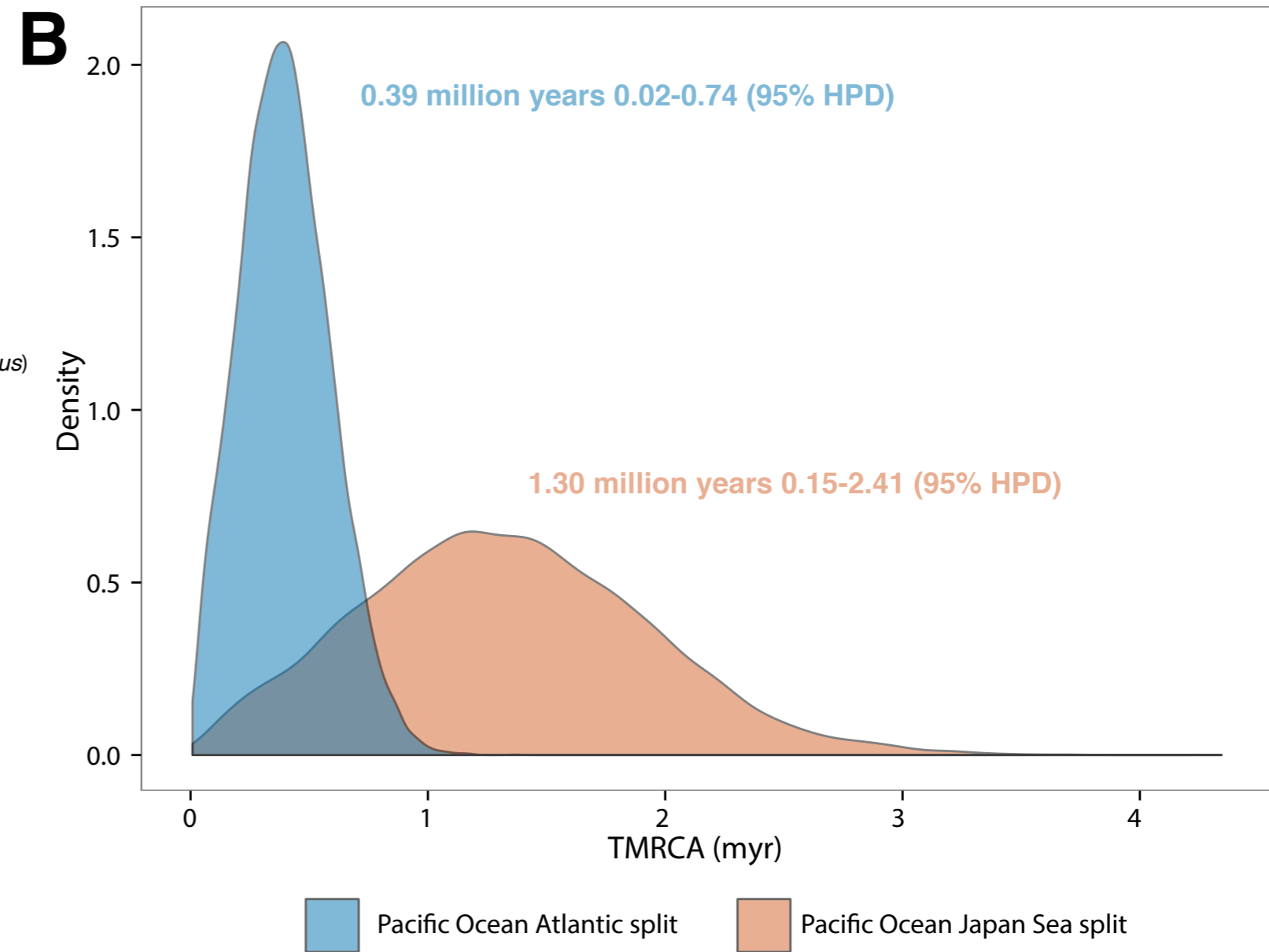

Supplement: S2 Fig — (A) Mitogenome Bayesian tree shows divergence between two mitochondrial clades–the Transpacific and European North American; asterisks on nodes indicate appropriate densities shown in (B). (B) Posterior probability densities for mitochondrial divergence time between G. aculeatus and G. nipponicus (pink) and between Pacific and Atlantic populations of the European North American clade (blue). (PDF) [file pgen.1007358.s009.pdf]

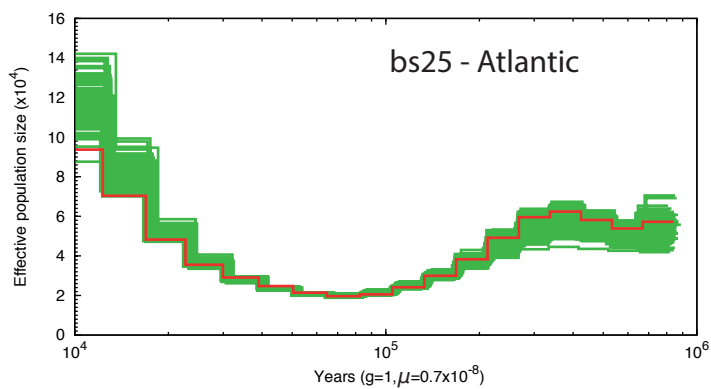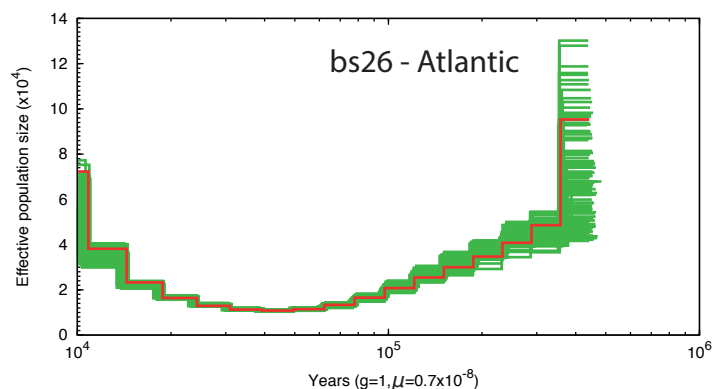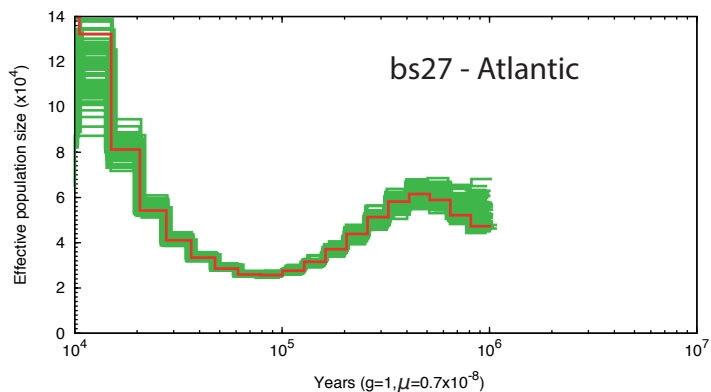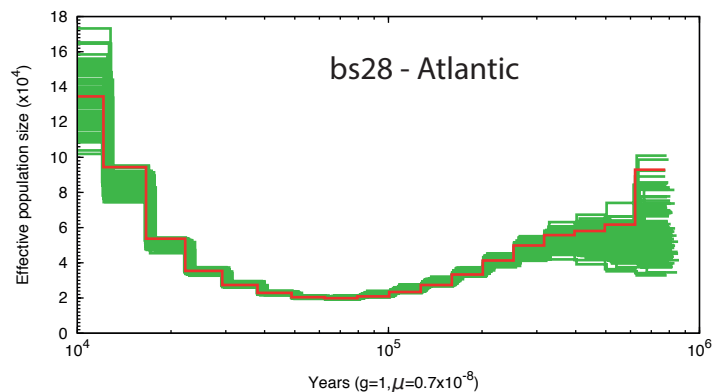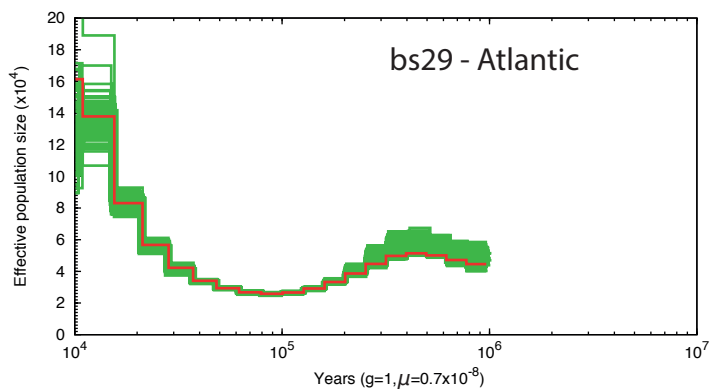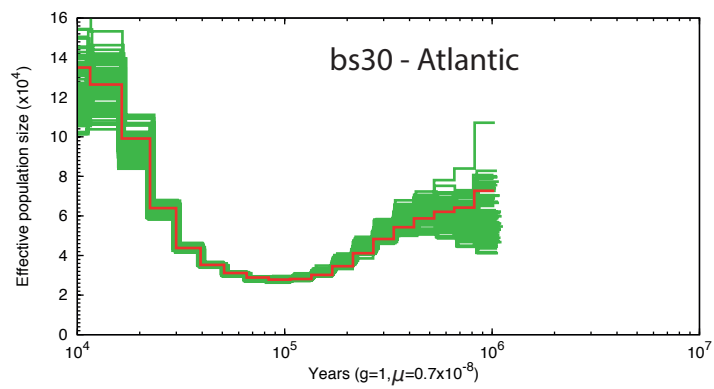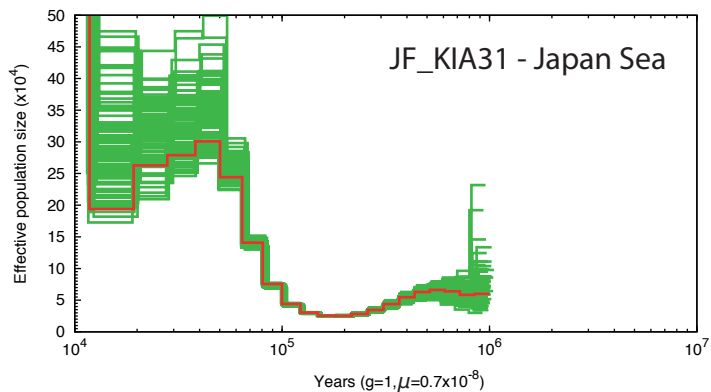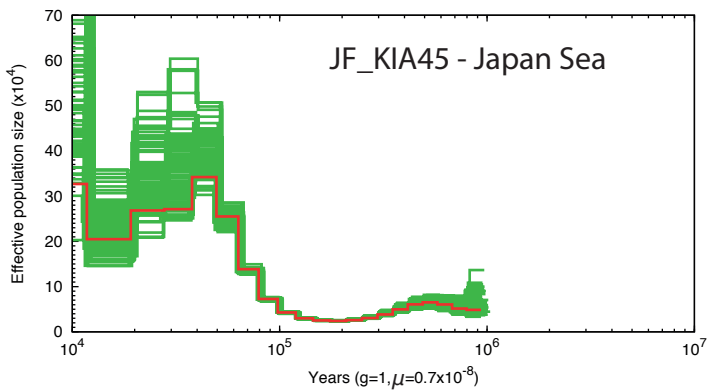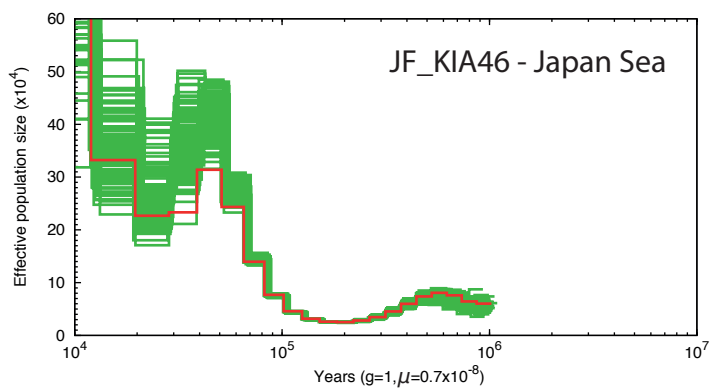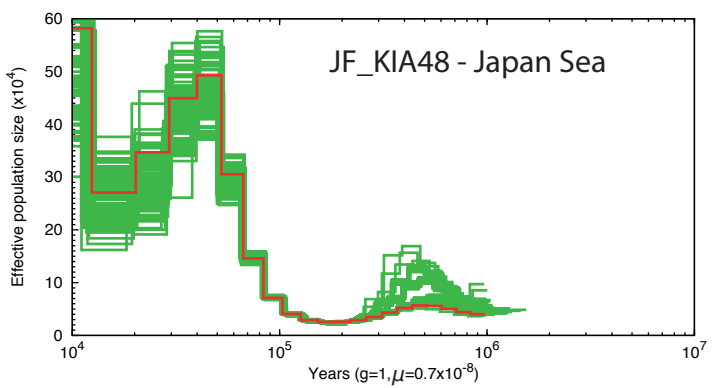

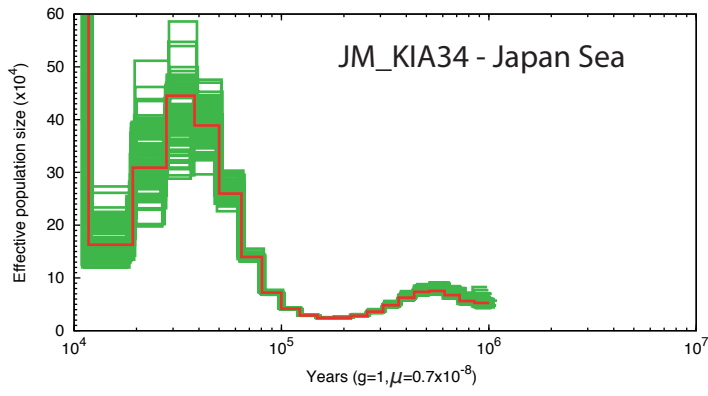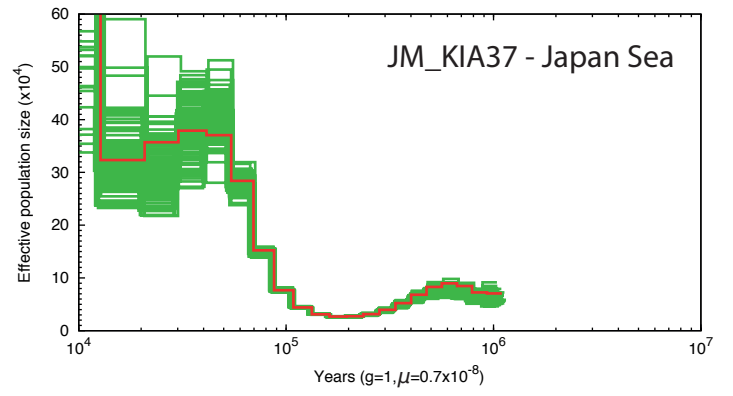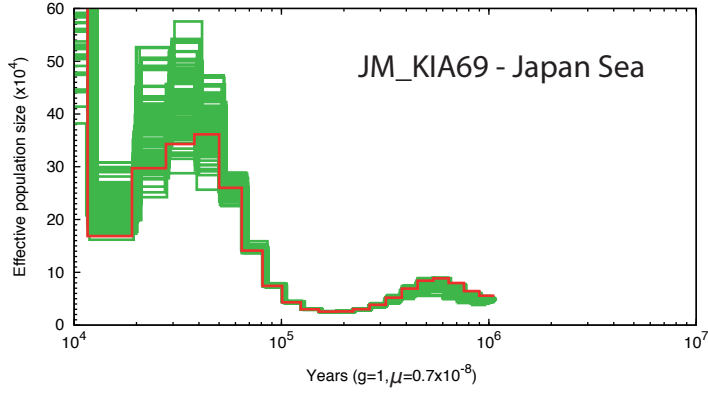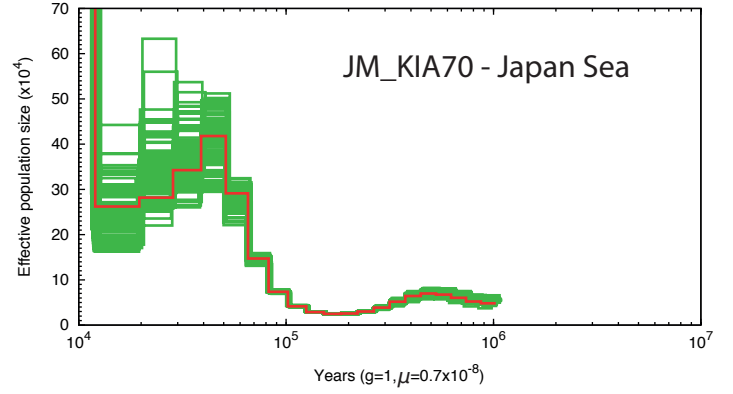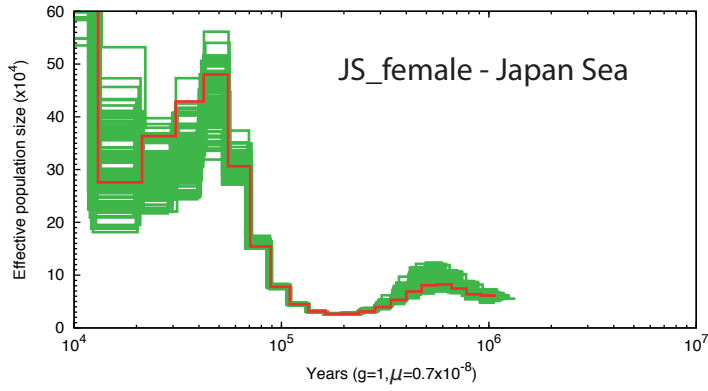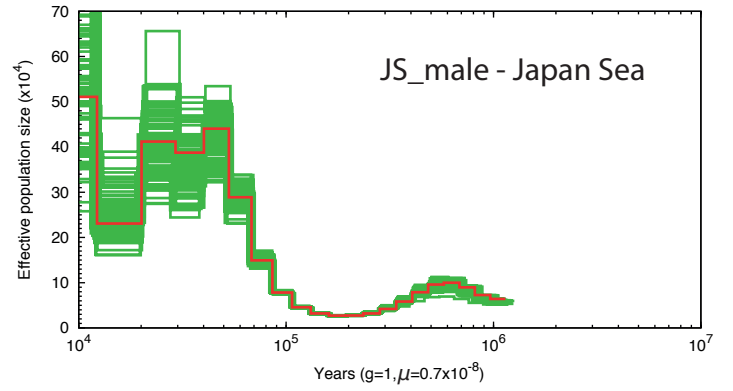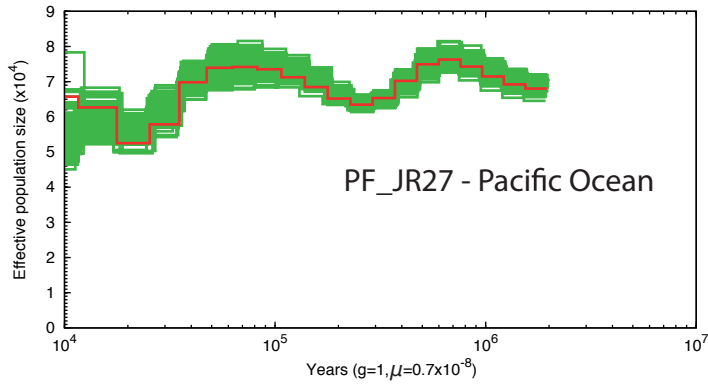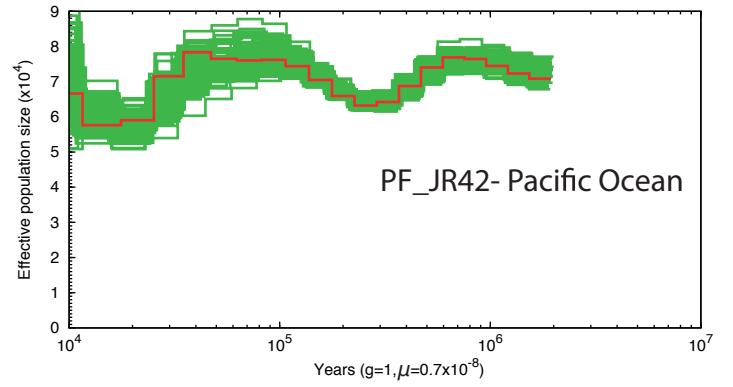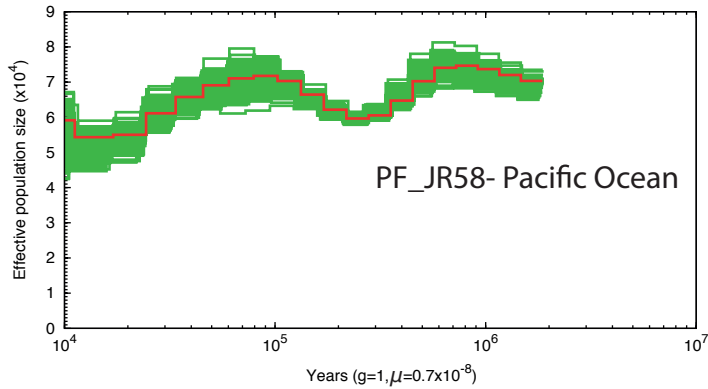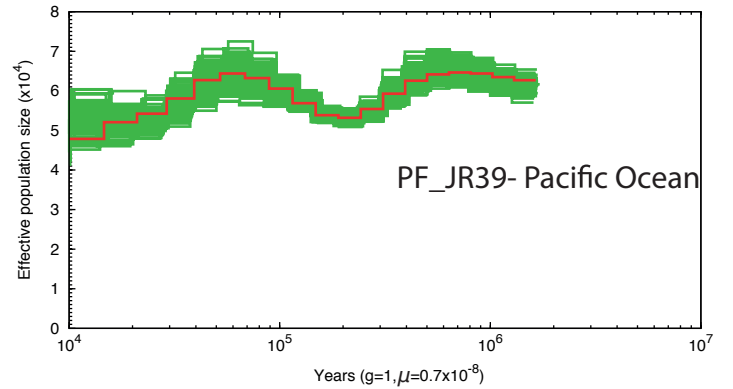

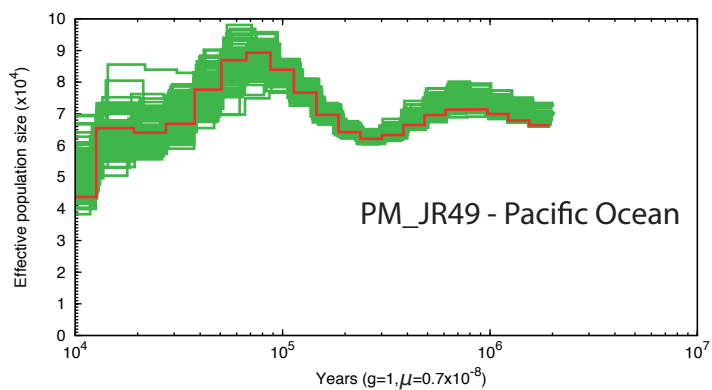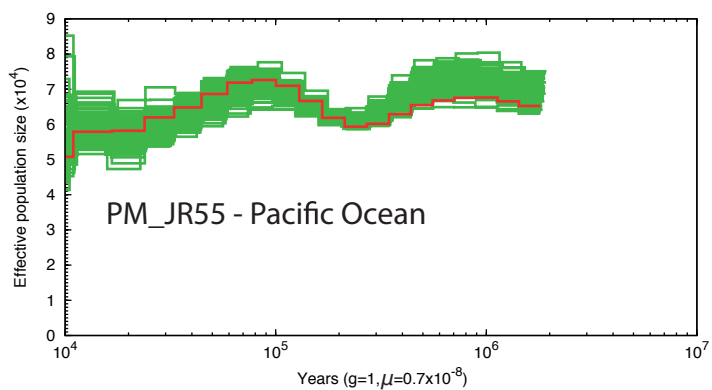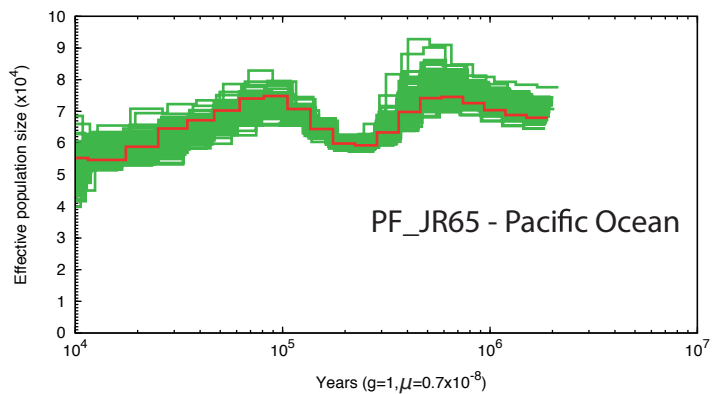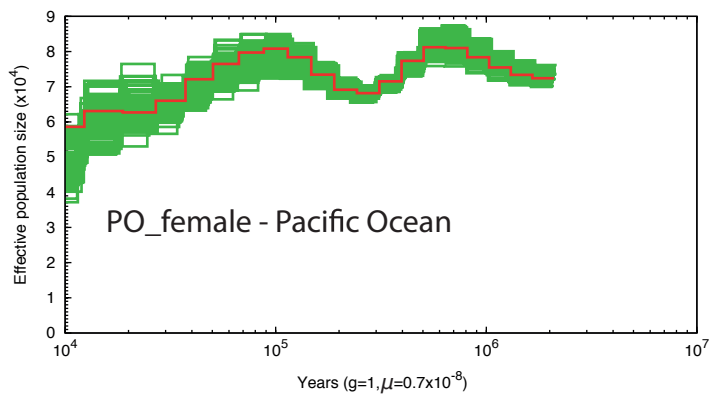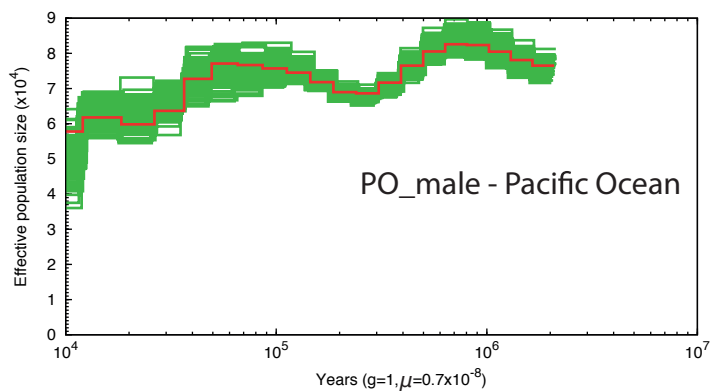

Supplement: S3 Fig — (PDF) [file pgen.1007358.s010.pdf]

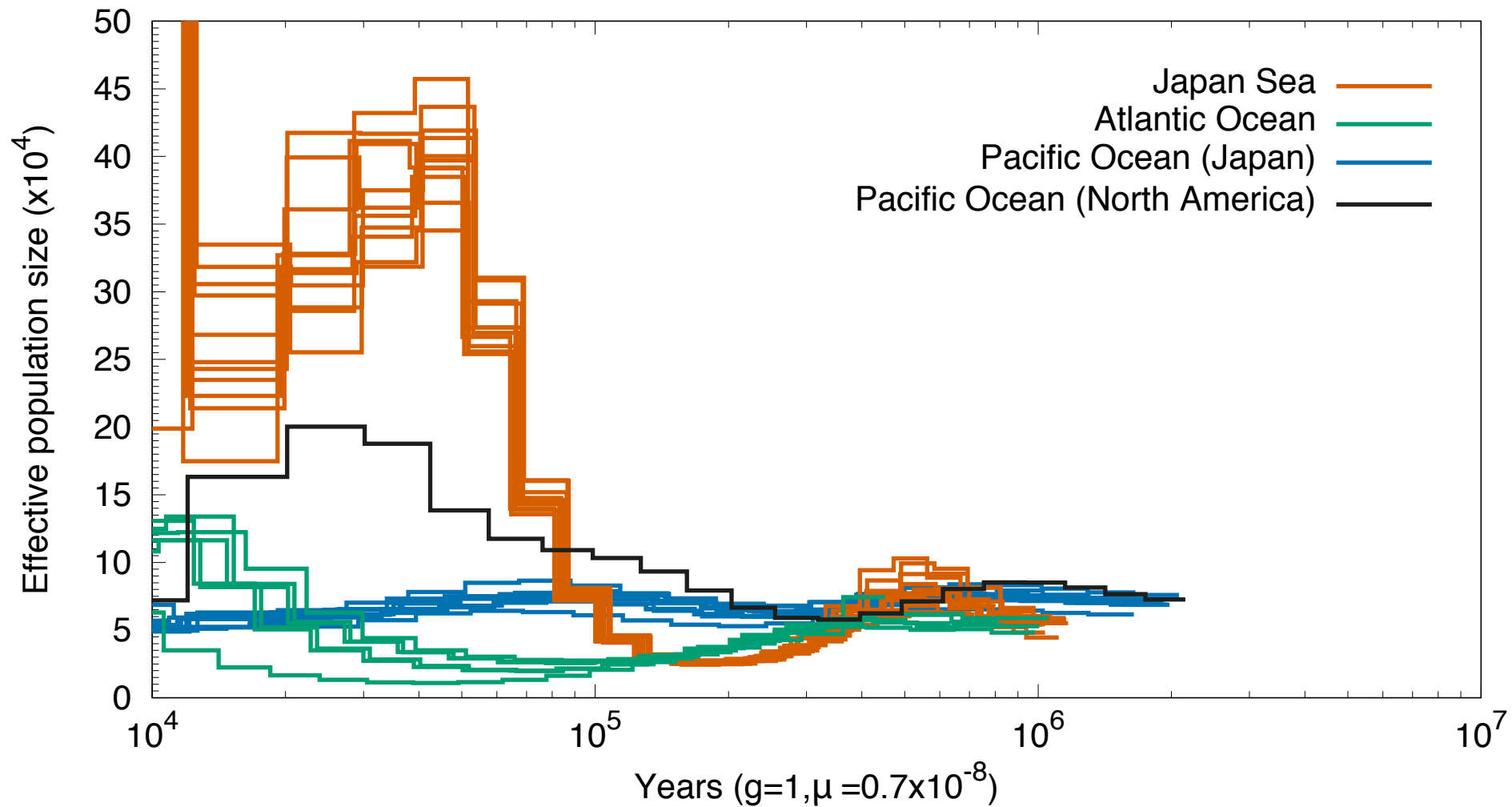

Supplement: S4 Fig — (PDF) [file pgen.1007358.s011.pdf]

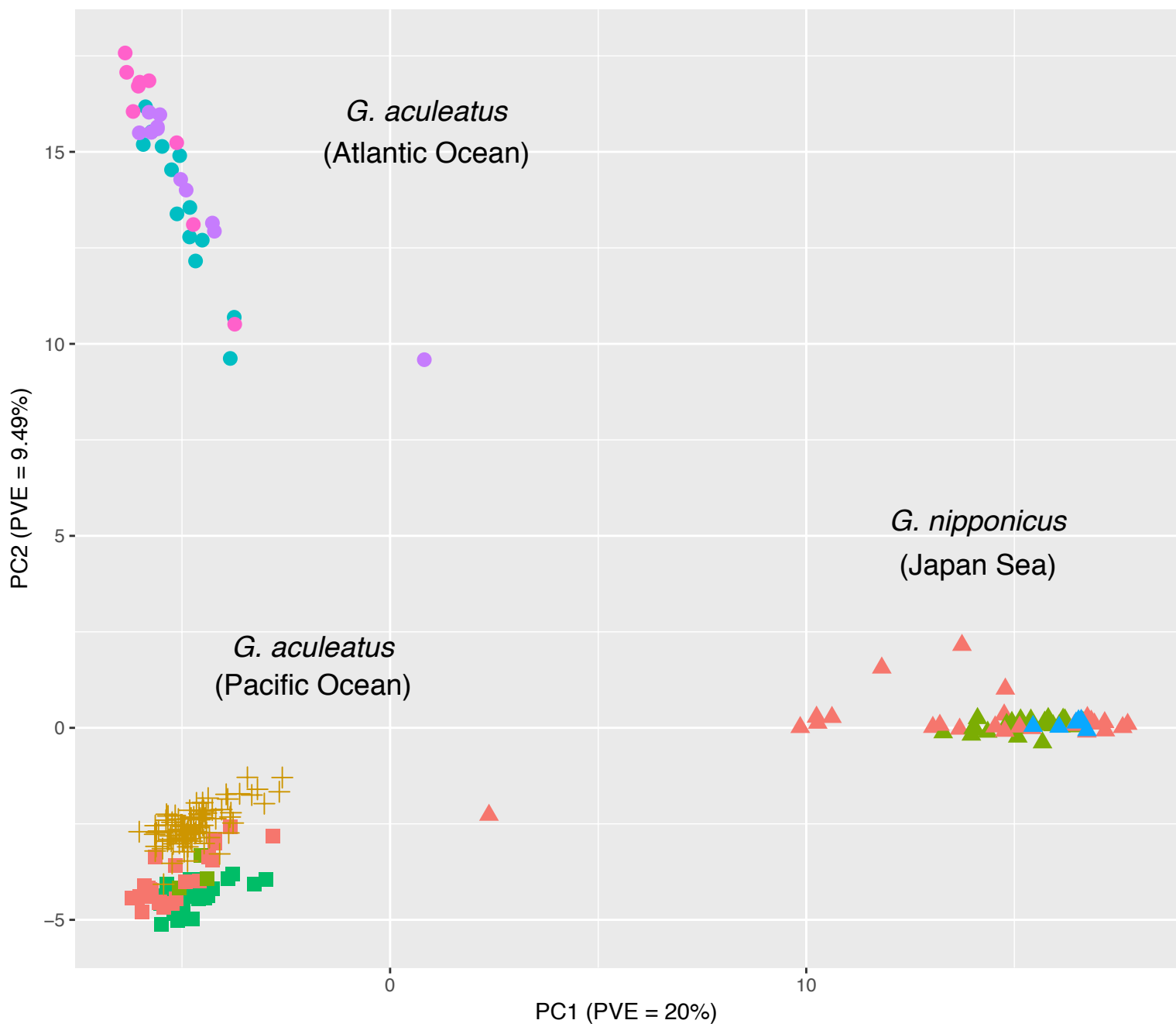

Supplement: S5 Fig — The arrow indicates the presence of an admixed individual occurring in the Akkeshi system. (PDF) [file pgen.1007358.s012.pdf]

**A**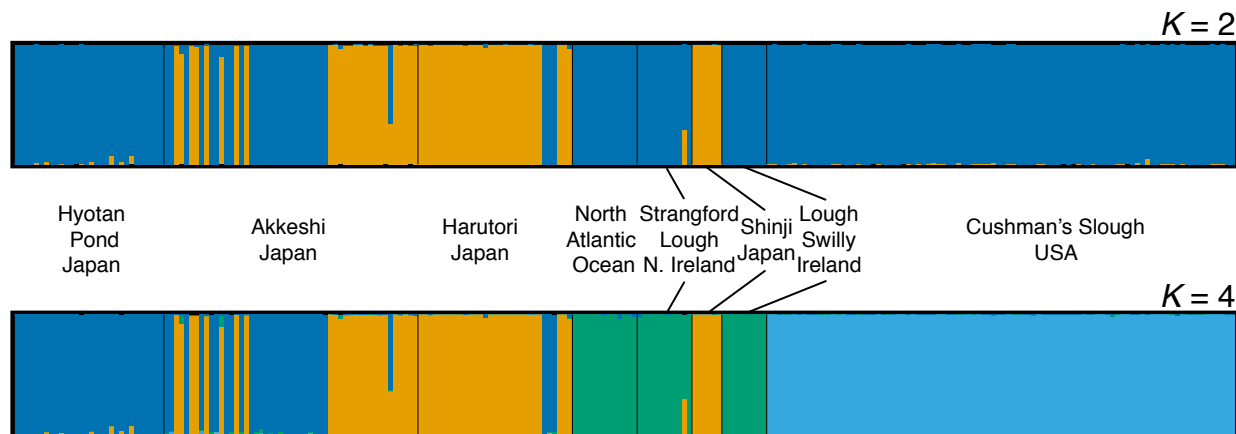**B**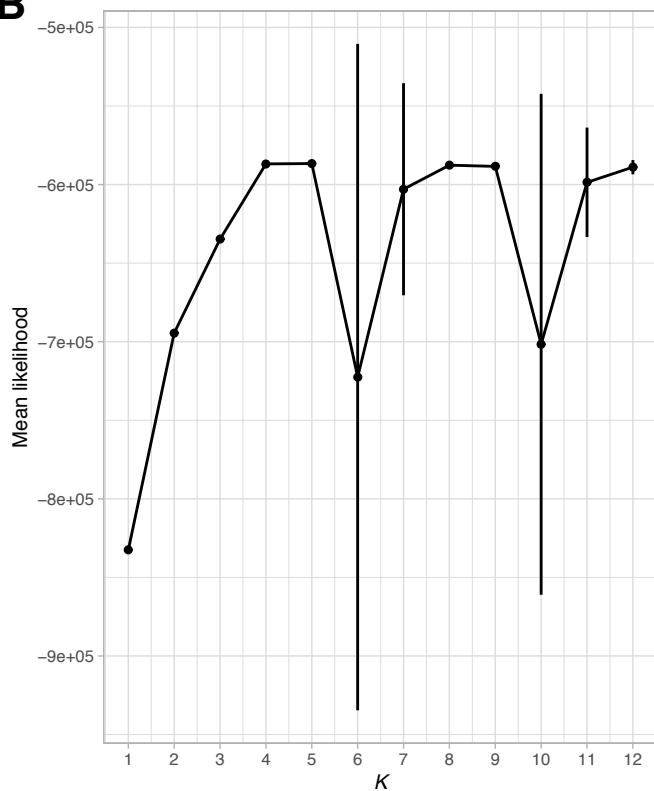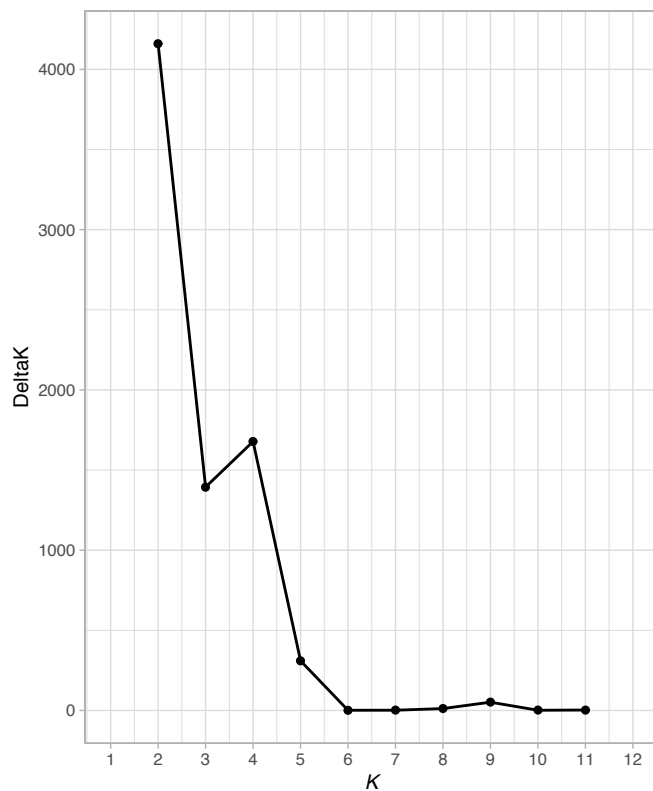

Supplement: S6 Fig — Analysis with K = 2 & 4 clusters (A), which is supported by likelihood analysis (B), showed the presence of admixed individuals in the Akkeshi system. (PDF) [file pgen.1007358.s013.pdf]

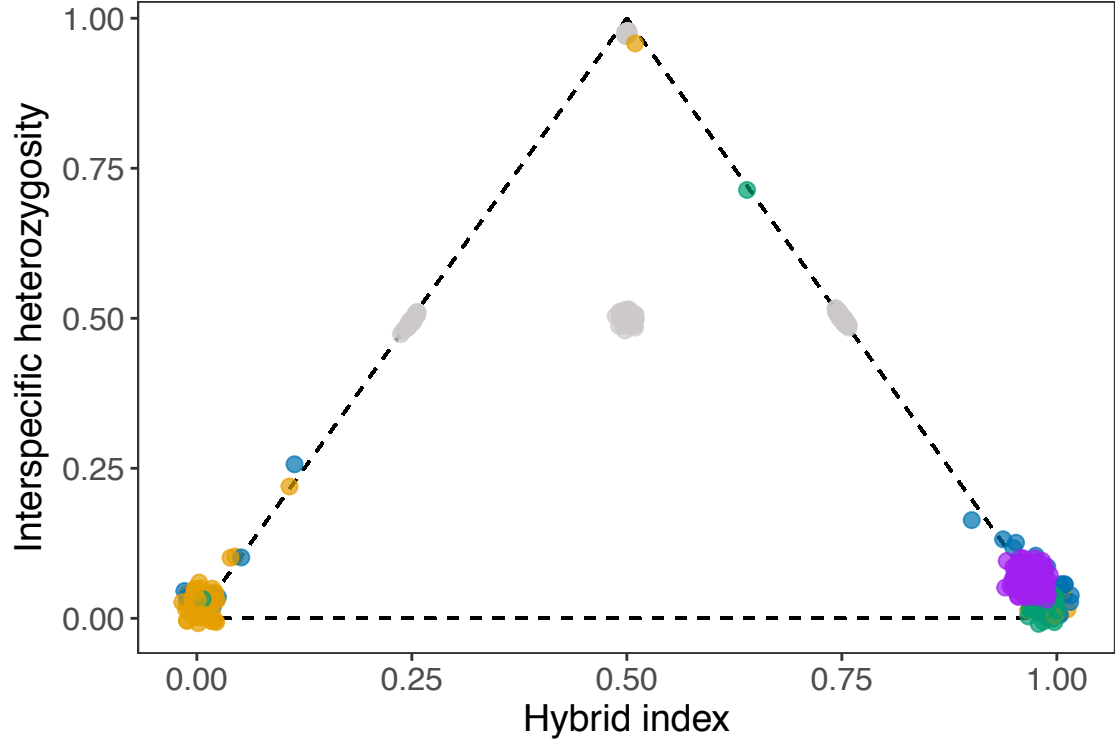

● Atlantic ● Japan Sea ● Pacific Ocean (Japan) ● Pacific Ocean (North America) ● Simulated hybrids

Supplement: S7 Fig — (PDF) [file pgen.1007358.s014.pdf]

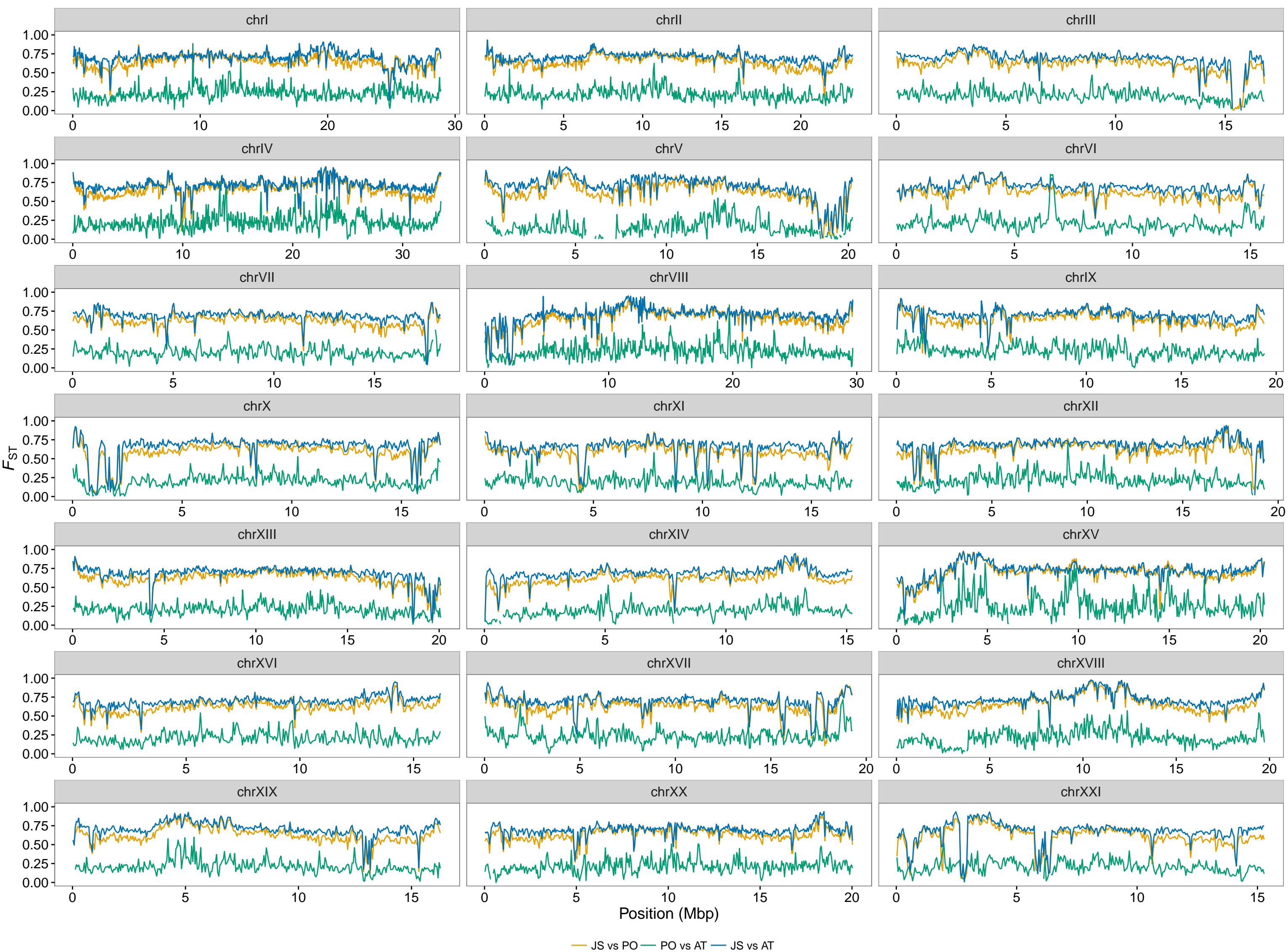

Supplement: S8 Fig — (PDF) [file pgen.1007358.s015.pdf]

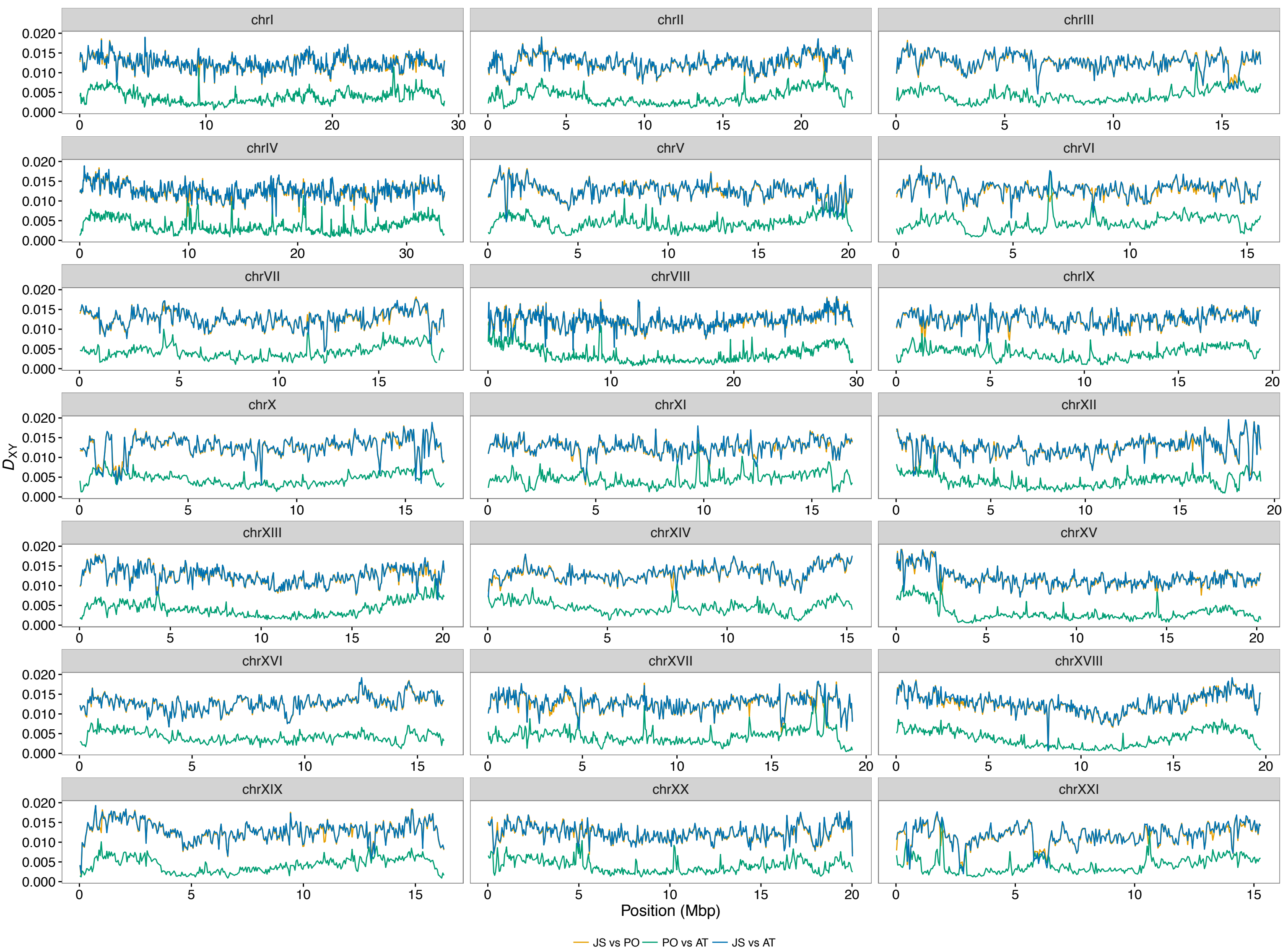

Supplement: S9 Fig — (PDF) [file pgen.1007358.s016.pdf]

JS v PO

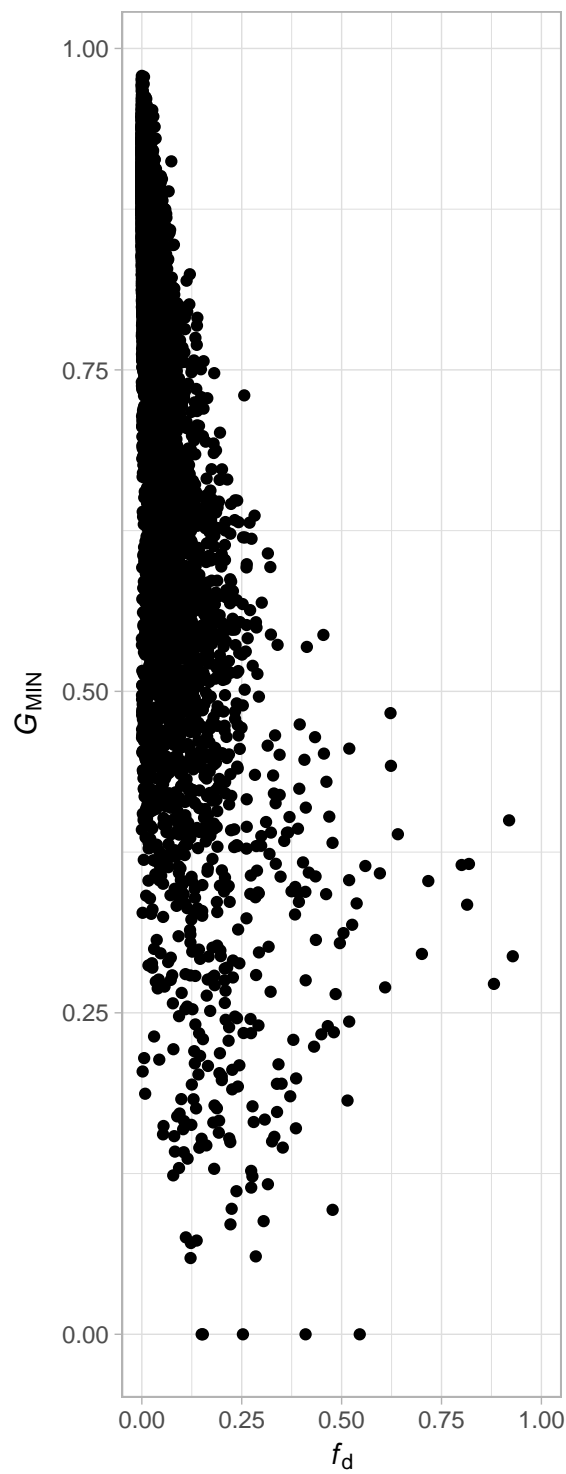

JS v AT

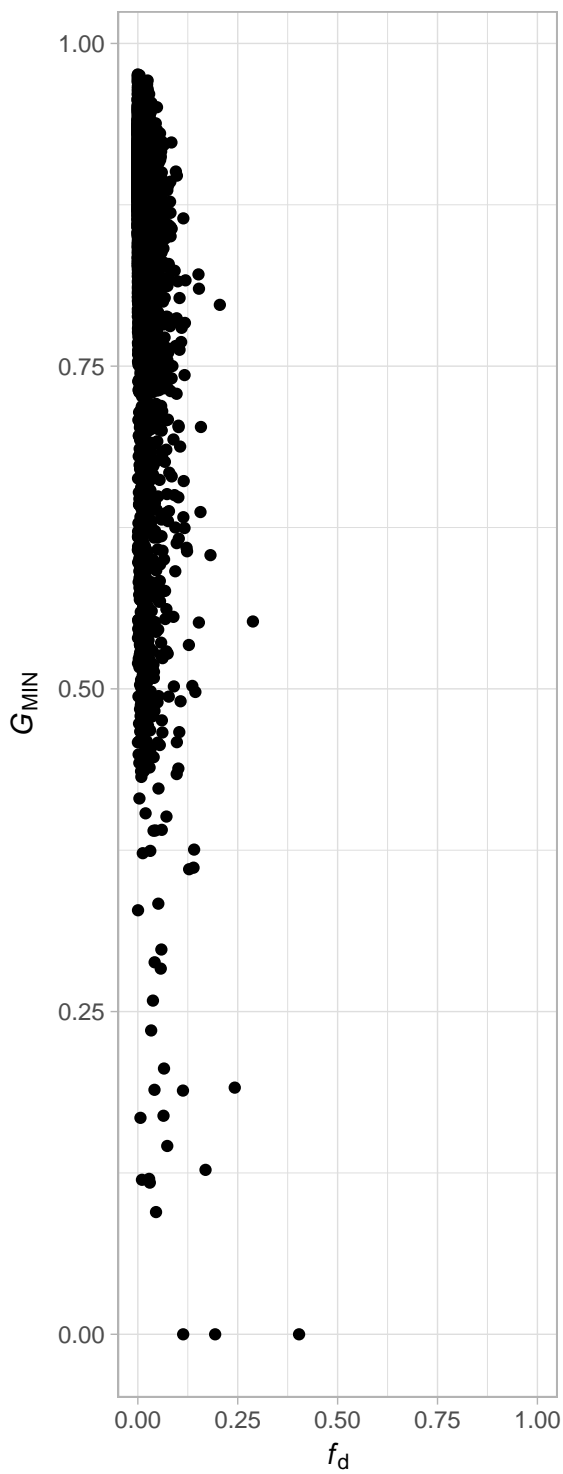

Supplement: S10 Fig — (PDF) [file pgen.1007358.s017.pdf]

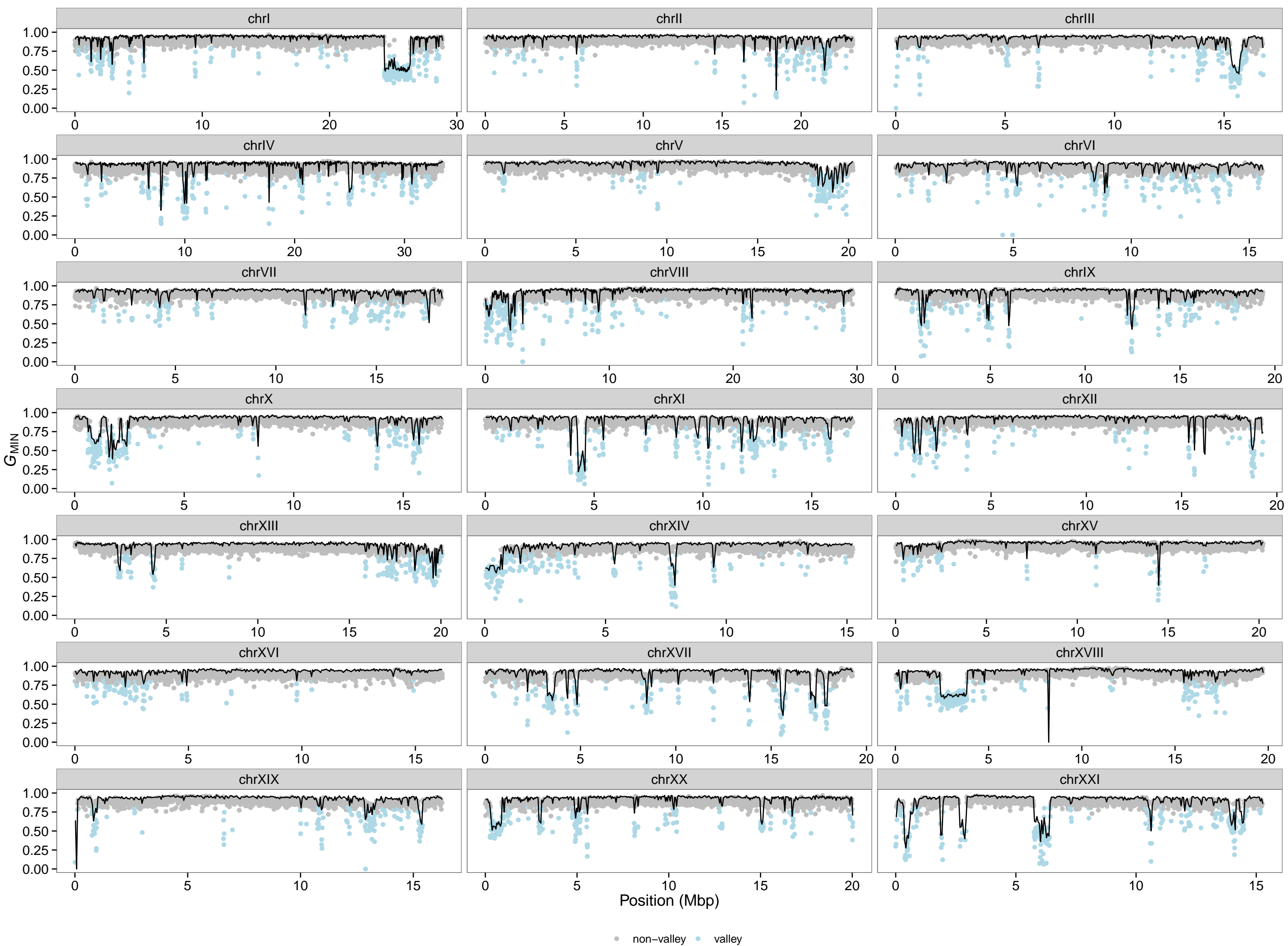

Supplement: S11 Fig — Black line represents 50 kb non-overlapping window GMIN signature. Points represent 10 kb windows; grey points are non-valley windows, blue points are valley windows identified by Hidden Markov Model algorithm. (PDF) [file pgen.1007358.s018.pdf]

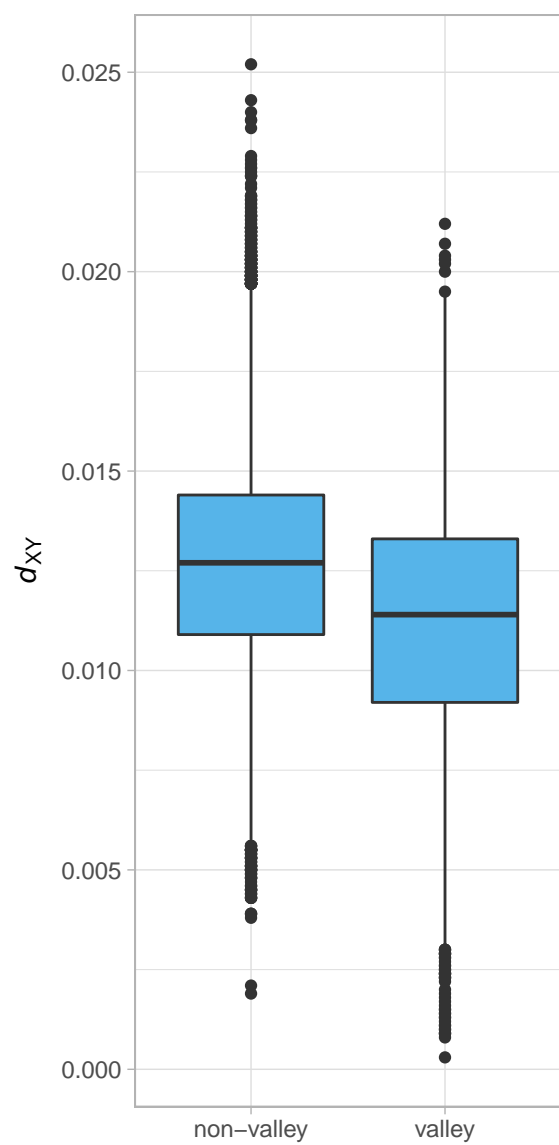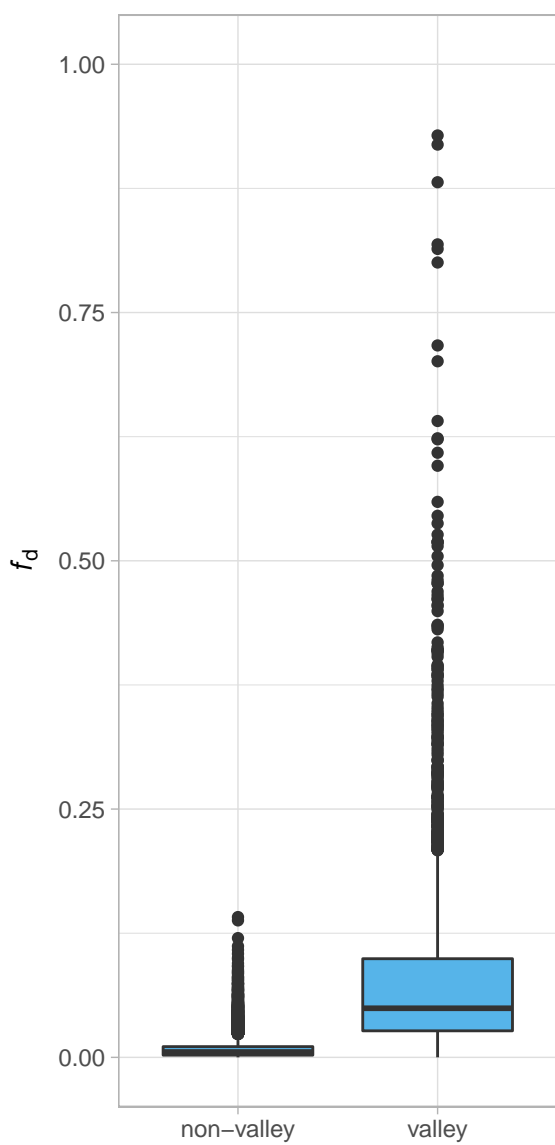

Supplement: S12 Fig — (PDF) [file pgen.1007358.s019.pdf]

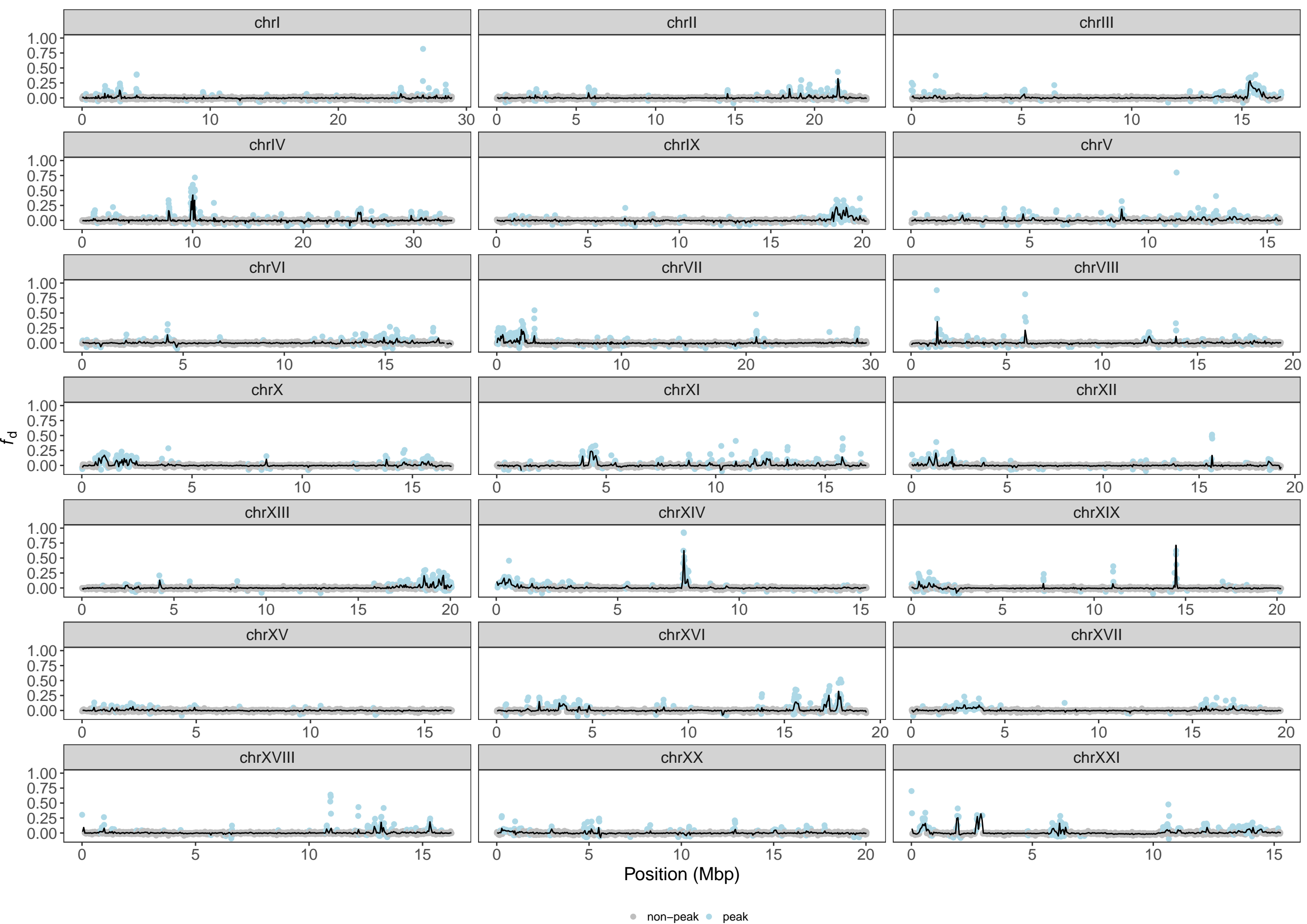

Supplement: S13 Fig — Black line represents 50 kb non-overlapping window fd signature. Points represent 10 kb windows; grey points are non-valley windows, while blue points are peak windows identified by Hidden Markov Model algorithm. (PDF) [file pgen.1007358.s020.pdf]

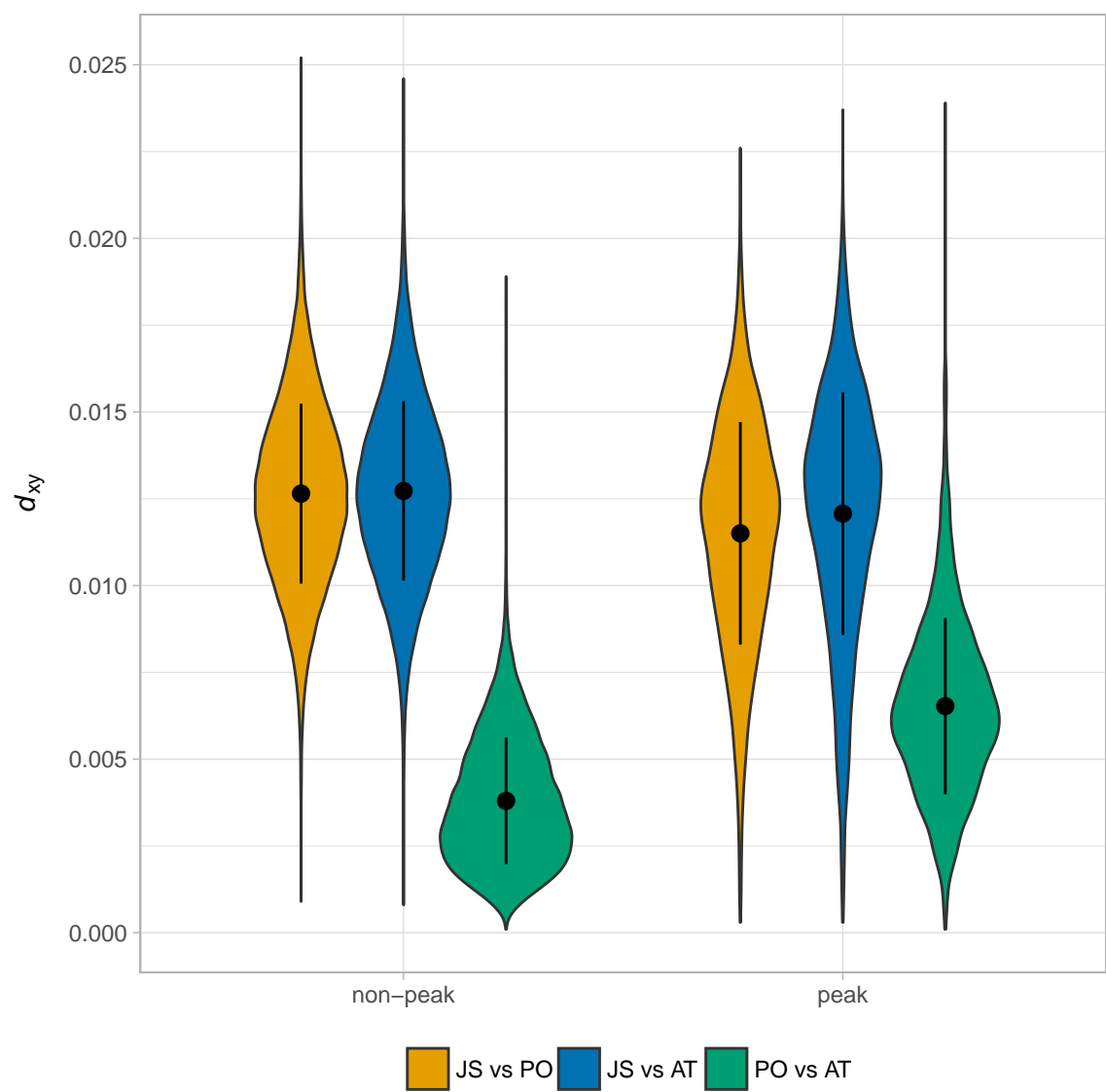

Supplement: S14 Fig — (PDF) [file pgen.1007358.s021.pdf]

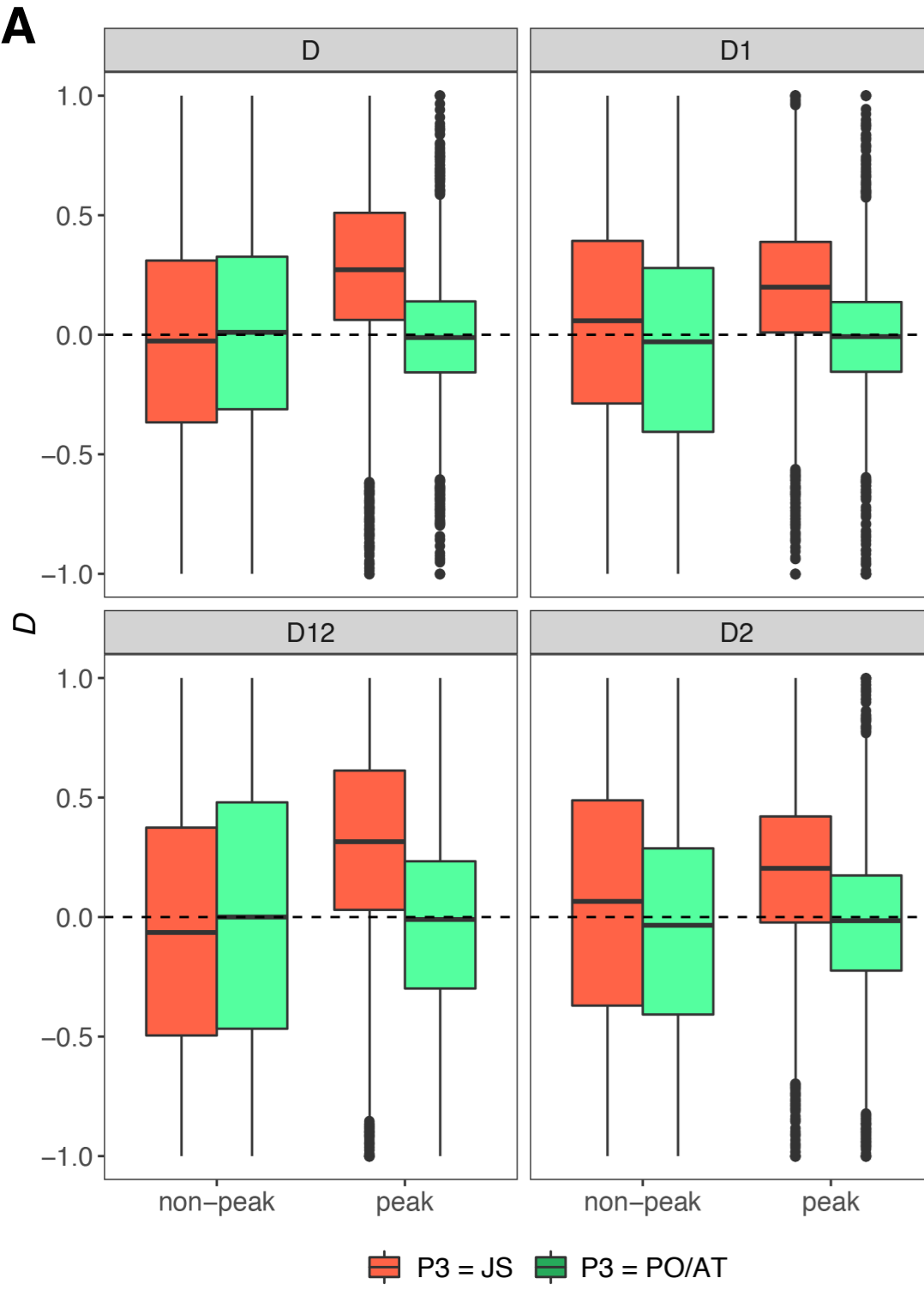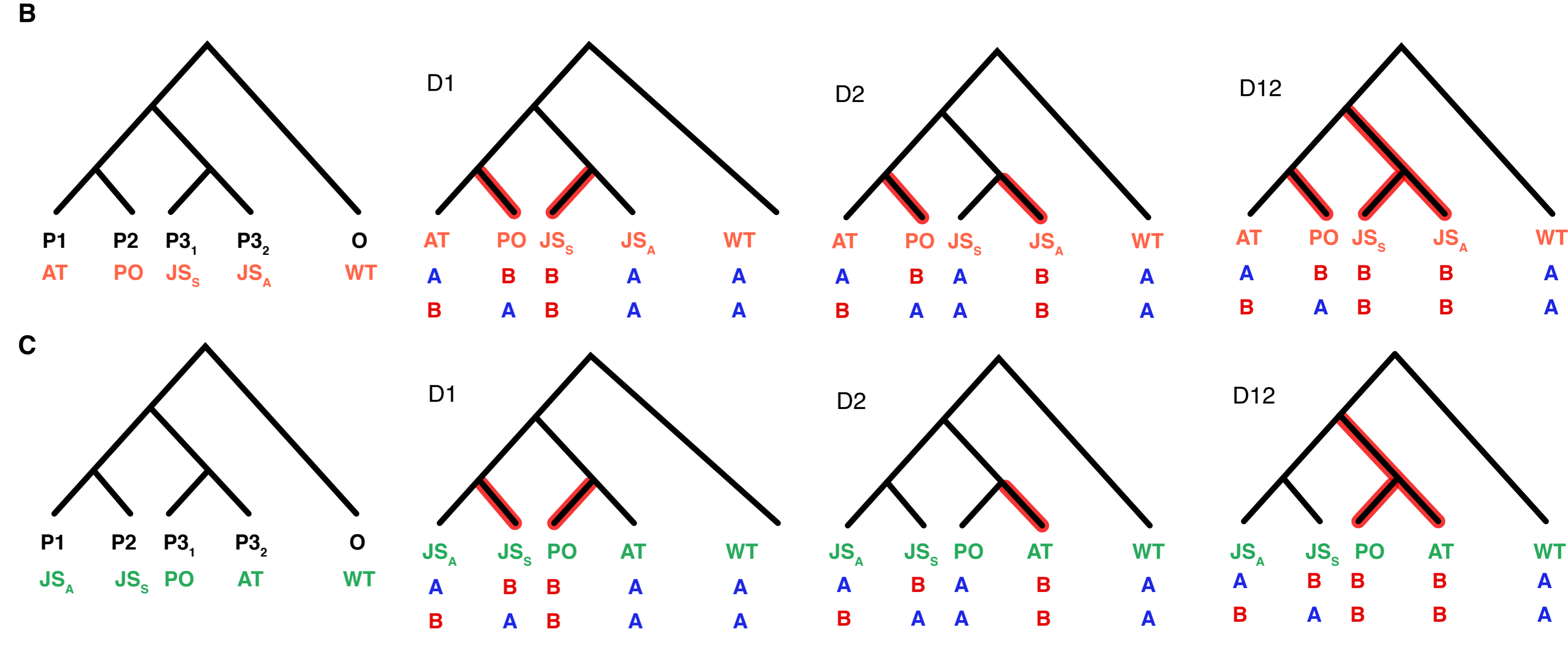

Supplement: S15 Fig — (A) Boxplots comparing partitioned D statistics between fd peaks and the autosomal background. Dashed line at zero indicates a balance between allele patterns indicative of incomplete lineage sorting. In (B), P1 = Atlantic Ocean (AT), P2 = Pacific Ocean (PO), P31 = sympatric Japan Sea (JSS), P32 = allopatric Japan Sea (JSA), and O = G. wheatlandi (WT). In (C), P12 and P3 were swapped. D1 measures asymmetry between P1 and P2 where the derived allele B is present in P31 but not P32, D2 measures where allele B is present in P32 but not P31, and D12 measures where the derived allele is shared by both P31 and P32. If we assume that the derived allele B occurred at the ancestor of P3, D12 indicates introgression from P3 to P2. See [61] for a more detailed explanation of these statistics. (PDF) [file pgen.1007358.s022.pdf]

$D_{\text{FOIL}}$

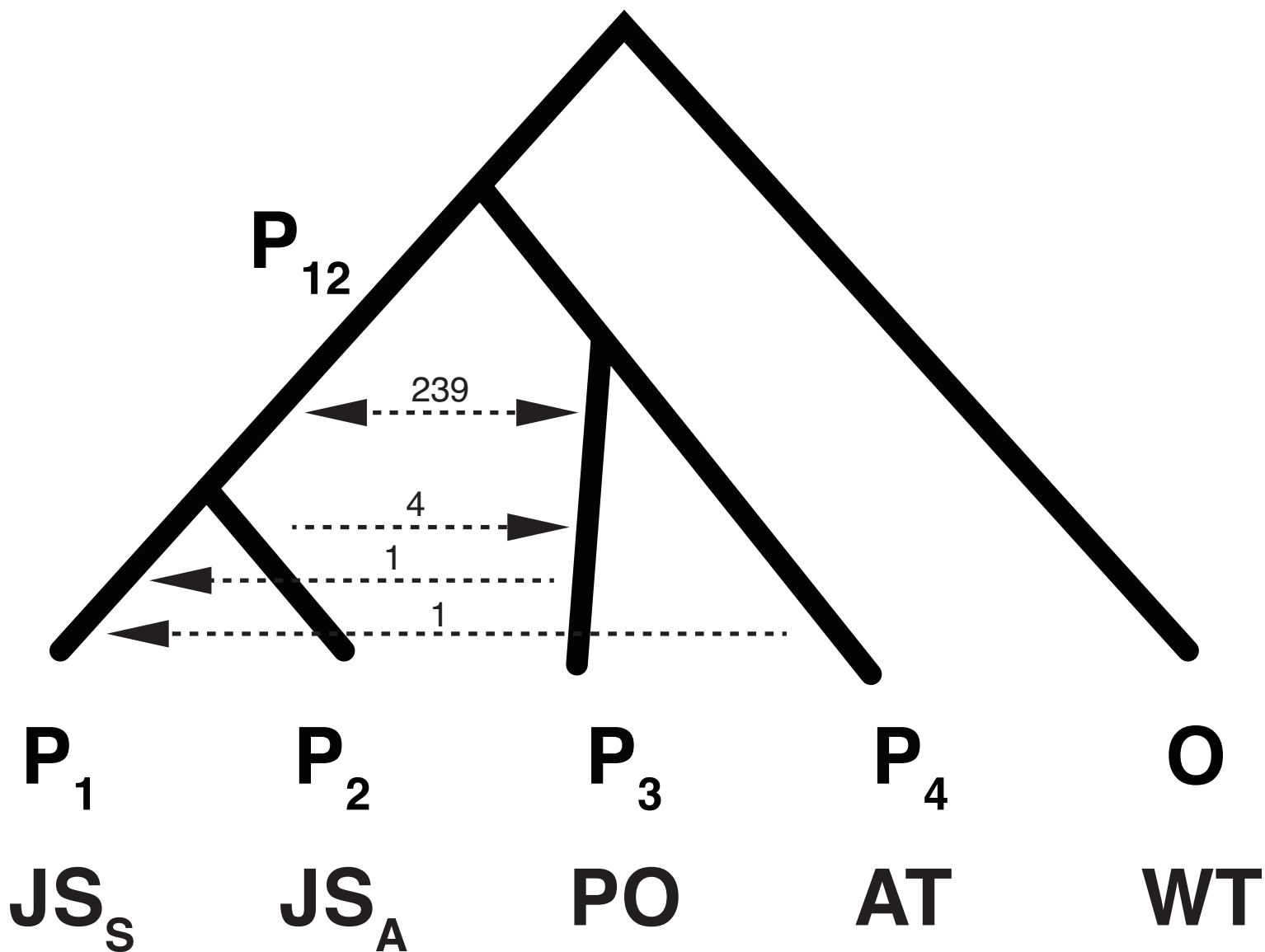

Supplement: S16 Fig — Using these statistics, we assume that the divergence time between P1 and P2 is younger than that between P3 and P4 and infer all possible introgressions including ancestral introgression involving P12. The number of loci (100 kb-window) that show statistically significant introgression are shown. See [62] for a more detailed explanation of these statistics. (PDF) [file pgen.1007358.s023.pdf]

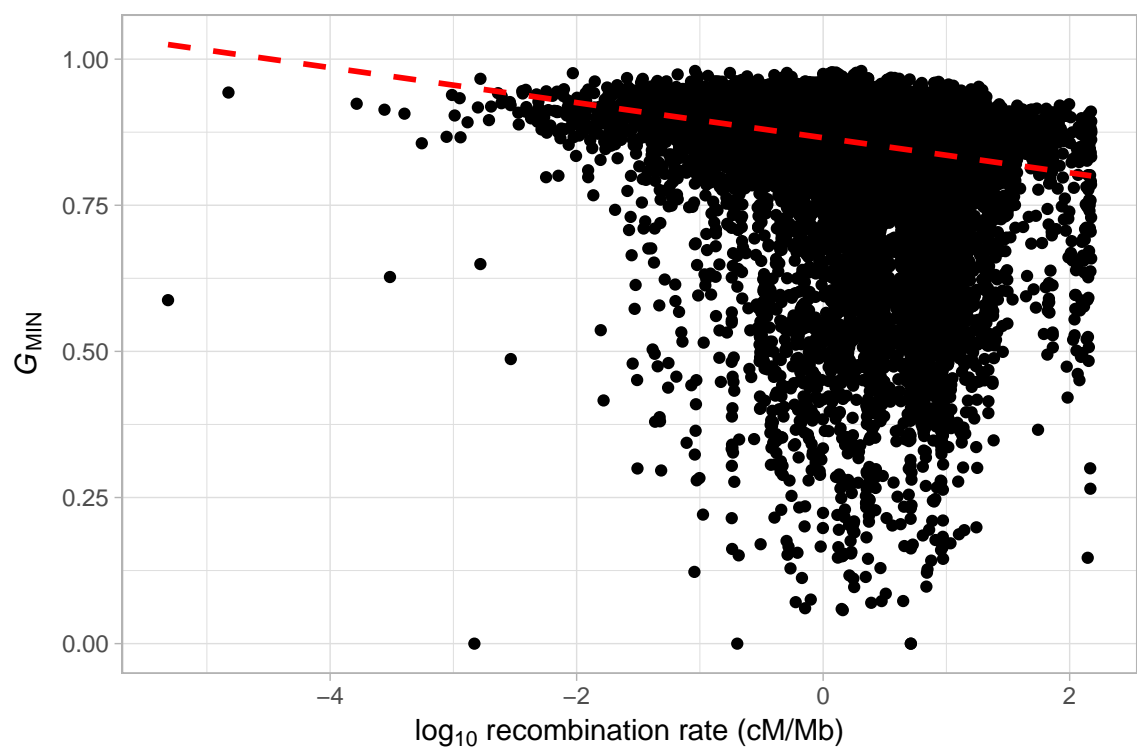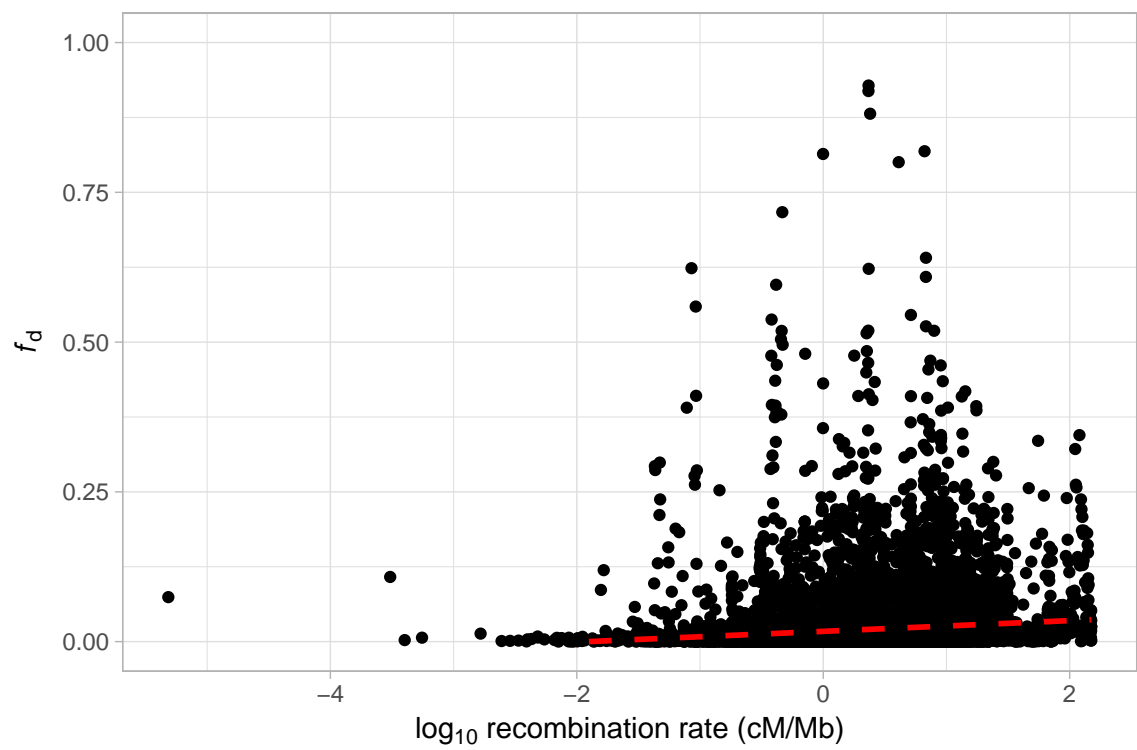

Supplement: S17 Fig — (PDF) [file pgen.1007358.s024.pdf]

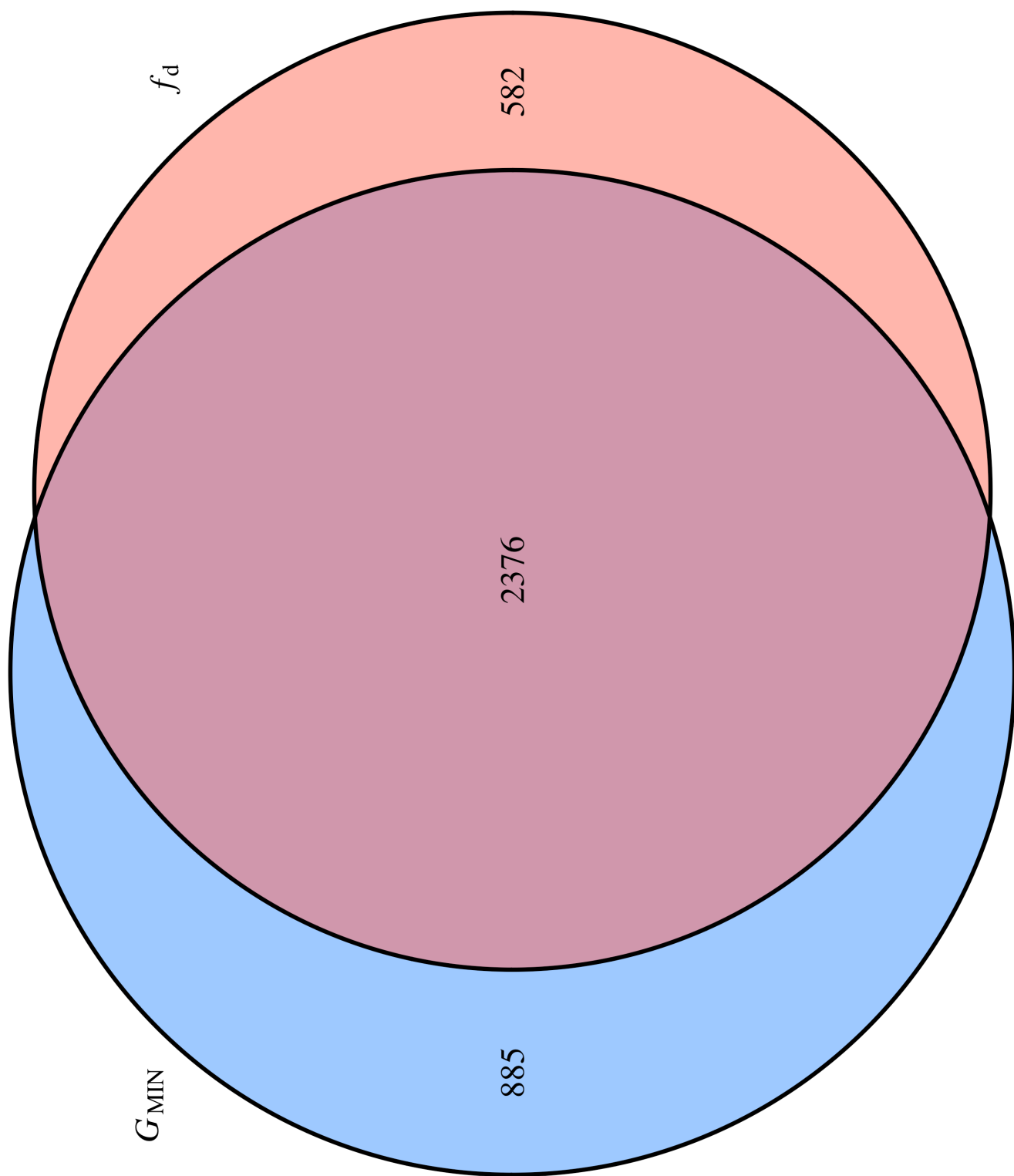

Supplement: S18 Fig — (PDF) [file pgen.1007358.s025.pdf]
