# Supplementary material for: The genomic landscape at a late stage of stickleback speciation: High genomic divergence interspersed by small localized regions of introgression
Source: PLoS Genet. 2018 May 23;14(5):e1007358. doi: 10.1371/journal.pgen.1007358 (PMC5988309; doi:10.1371/journal.pgen.1007358)
Supplement: S1 Text — (PDF) [file pgen.1007358.s026.pdf]

Supplementary methods & results

Mark Ravinet, Kohta Yoshida, Shuji Shigenobu, Atsushi Toyoda, Asao Fujiyama and Jun Kitano

26th March 2018

Approximate Bayesian Computation

In order to test different divergence hypotheses between *G. aculeatus* and *G. nipponicus*, we used Approximate Bayesian Computation (ABC). This flexible estimation framework allowed us to evaluate posterior probability values amongst models and also to estimate demographic parameters under each divergence scenario.

Sampling observed data

For population genomic data, full sequence loci provide highly accurate estimation using ABC (Robinson et al. 2014). In order to obtain loci suitable for our ABC analysis, we randomly sampled putatively neutral nuclear loci from across the genome. Using a custom R script we produced a BED file of reference genome coordinates for 2 kb loci randomly sampled at 125 kb intervals; resulting in 2378 potential loci for each of the 20 individuals included in the analysis (i.e. *G. nipponicus* and Pacific *G. aculeatus only*). The consensus sequence from each locus was then called from our consensus vcf using a custom `python` script. This script created two haplotypes for each locus, randomly assigning heterozygous variants to one of the two haplotypes to account for unphased data (Robinson et al. 2014). Loci were then screened to include only those occurring on autosomes, > 1000 bp sequence and a minimum of 70% coverage (i.e. > 70% bases called within a locus) for all 20 individuals. This resulted in a final dataset of 1874 loci. Functions and scripts for generating coordinates and extracting and filtering consensus sequences are available on GitHub (https://github.com/markravinet/genome_sampler).

Divergence models with a hierarchical approach

To test our hypotheses on the evolutionary history of *G. aculeatus* and *G. nipponicus*, we constructed five divergence models – isolation (I), isolation with migration, isolation-with-ancient-migration (IAM), isolation-with-recent-migration (IRM) and isolation-with-ancient-and-recent-migration (IARM; see Fig A1 and Table A1 for a list of parameters estimated in each model). Since the results of our PSMC analyses clearly indicate effective population size has varied throughout divergence (Fig 1D in main text), we also constructed three population size change models – constant population size, population growth, and a bottleneck in *G. nipponicus* following divergence (see Fig A1 and Table A2). With three population size models for each of the five divergence scenarios, a total of 15 demographic models were tested.

With the exception of I, all models incorporated gene flow. Assuming a uniform migration rate when genome-wide migration is heterogeneous (as is the case in this system) can significantly impair the accuracy of ABC approaches (Roux et al. 2013; Roux et al. 2014). To account for this, we allowed heterogeneous gene flow by drawing per-locus migration parameters from a scaled β distribution defined by α, β and a scalar, c (Roux et al. 2013; Roux et al. 2014). Prior distributions for all parameters are shown in Tables A1 and A2. As is required when using `ms`, all parameters for the simulation step were scaled to an arbitrary reference population size – N0 – set to 50,000 here.

To properly evaluate demographic models, we used a hierarchical ABC approach (Nadachowska-Brzyska et al. 2013; Fagundes et al. 2007). We first performed a model selection step on the different population size models within each divergence scenario. Then using the best-supported population size model for each

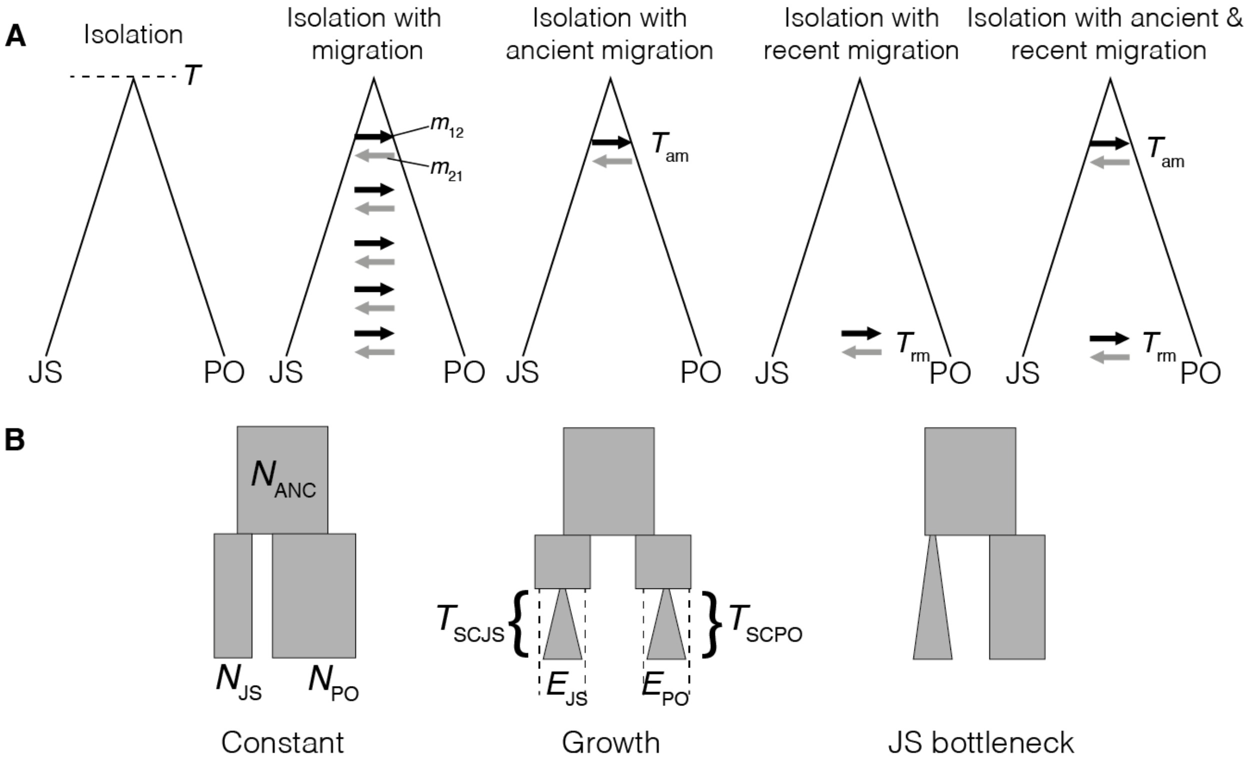

Figure A1: Models used for Approximate Bayesian Computation. (A) main divergence scenarios and (B) population growth models nested within divergence scenarios.

divergence scenario, we performed a final round of model selection to determine which of the five divergence hypotheses best fit our observed data.

Simulation step

For each of the 15 models, we simulated 1 x 10^6 datasets, resulting in a total of 15 million simulations. Following Robinson et al. (2014), we used a custom `R` based control script and `msABC` (Pavlidis, Laurent, and Stephan 2010) to perform simulations, calculate summary statistics and quantify their distribution across the genome in a single step. This approach offers considerable flexibility in establishing prior probability distributions for each of the estimated parameters. Furthermore given the large size of our dataset (i.e. ~1900 loci for 20 individuals) each simulation produces a large amount of data, making storage a challenge. Using `R` to interface with `msABC` allowed us to greatly reduce the required data storage. Initial calculations suggested that for the most complex models, 1 x 10^6 simulations would take over 365 days to complete in a single run. Therefore we used a combination of `GNU Parallel` (Tange 2011) and independent runs across multiple computing cores to reduce analysis speed to just 2 days per model (scripts and additional instructions available on Github: https://github.com/markravinet).

Sensitivity analysis using pseudo-observed datasets

Before beginning this model selection step, we performed a thorough sensitivity analysis to investigate the efficacy of our ABC approach. This sensitivity analysis had two main aims; 1) to test the ability of our ABC framework to successfully distinguish different demographic models and 2) to evaluate the reliability of our demographic parameter estimates under each model. In order to perform this investigation of our ABC framework, we used pseudo-observed datasets (PODs). PODs are generated by randomly selecting a

	Description	Prior	Models
θ	Scaled mutation rate	0.2-40, uniform	All
T	Divergence time	Mean = 1.5 myr, SD = 0.8, lognormal	All
m_{12}	Migration rate PO>JS	rate = 0.1, exponential	IM, IAM, IRM, IARM
m_{21}	Migration rate JS>PO	rate = 0.1, exponential	IM, IAM, IRM, IARM
T_{am}	Timing of ancient migration	T/2 - T, uniform	IAM, IARM
T_{rm}	Timing of recent migration	0 - T/2, uniform	IRM, IARM
α	Hyperprior - α parameter	0-5, uniform	Heterogenous migration models
β	Hyperprior - β parameter	0-200, uniform	Heterogenous migration models
c_1	Scalar for β distribution	0-15, uniform	Heterogenous migration models
c_2	Scalar for β distribution	0-15, uniform	Heterogenous migration models

Table A1: Parameters and priors used in divergence scenarios for ABC estimation. N.B. Migration parameters are expressed backwards in time. Abbreviations are: IM – isolation with migration, IAM – isolation with ancient migration, IRM – isolation with recent migration, IARM – isolation with ancient and recent migration.

Symbol	Description	Prior	Models
N_{JS}	JS population size	0-3, uniform	CV, SPG, RPG, JSB
N_{PO}	PO population size	0-3, uniform	CV, SPG, RPG, JSB
TSC_{JS}	Timing of JS growth	0-T/2, uniform	RPG, JSB
TSC_{PO}	Timing of PO growth	0-T/2, uniform	RPG, JSB
x	Fraction of population size	0-0.5, uniform	APG, RPG, JSB
E_{JS}	Exponential JS growth rate	$1/TSC_{JS}*\log(N_t/N_0)$†	APG, RPG, JSB
E_{PO}	Exponential PO growth rate	$1/TSC_{PO}*\log(N_t/N_0)$†	APG, RPG, JSB

Table A2: Parameters and priors used in growth models for ABC estimation. Abbreviations are: CI – constant identical, CV – constant variation, APG – ancient population growth, RPG – recent population growth and JSB – Japan Sea bottleneck. †Where N_0 is derived from $4N_0$ and N_t is N_0*x

simulated dataset from the reference table and then rerunning estimation steps in the ABC pipeline using it as the observed dataset (hence pseudo-observed). Since PODs are generated under a known model with known parameters, they can be used to estimate the rate of selecting the true model in a model selection step and also the precision error on demographic parameter estimates.

To assess the accuracy of our hierarchical model selection we used the `cv4postpr` function from the `R abc` package to generate PODs. For each round of POD analysis we varied three parameters that influence model selection: the number of summary statistics, the rejection method used to accept simulated datasets close to the observed, and the tolerance level (i.e. the proportion of accepted simulated datasets). For each simulation, we generated a maximum of 29 summary statistics using `msABC` and then subsequently subsampled these summary statistics to examine the effects of dimensionality in the data; thus we tested analyses with 12, 20 and 29 summary statistics (see Table A3 for statistics used). Numerous methods exist for the rejection step of ABC and all perform differently depending on dimensionality in the data. To account for this, we used the simple rejection method, multinomial regression (Nielsen and Beaumont 2009) and a neural network approach (Blum and François 2010). Tolerance levels varying between 1-3% can also influence model selection and parameter estimation (Robinson et al. 2014). Therefore we performed all analyses using 0.01%, 0.05%, 1% or 3% tolerance to assess how this influenced model selection (i,e, 1,000, 5,000, 10,000 and 30,000 retained datasets respectively). For each round of model selection, 1000 PODs were estimated for each of the models assessed (the three growth models in the first hierarchical step and all five divergence scenarios in the second step) and the model with the highest posterior probability selected.

Each analysis round produced a POD confusion matrix - i.e. a matrix with rows representing each true model and columns representing the chosen model. From this we calculated proportion of PODs correctly identified as the true model (‘true rate’ hereafter) and the type I and type II error rates. Error rates estimated from

Statistic	ss29	ss20	ss12
Segregating sites PO	x	x	x
Segrating sites JS	x	x	x
Segrating sites both	x	x	x
Nucleotide diversity PO	x	x	x
Nucleotide diversity JS	x	x	x
Theta (π)	x	x	-
Watterson's Theta PO	x	x	-
Watterson's Theta JS	x	x	-
Watterson's Theta (both)	x	x	-
Tajima's D	x	x	x
Tajima's D PO	x	x	x
Tajima's D JS	x	x	x
ZnS PO	x	-	-
ZnS JS	x	-	-
ZnS both	x	-	-
PO v JS F_{ST}	x	x	x
Perc. Shared sites	x	x	x
Perc. Private sites	x	x	x
Perc. Fixed diffs	x	x	x
Fay & Wu's H PO	x	-	-
Fay & Wu's H JS	x	-	-
Fay & Wu's H both	x	-	-
Haplotype diversity PO	x	x	-
Haplotype diversity JS	x	x	-
Haplotype diversity both	x	x	-
Haplotype number PO	x	-	-
Haplotype number JS	x	-	-
Haplotype number	x	-	-

Table A3: Summary statistics used in each ABC analysis round.

PODs are analogous to false positive and false negative rates with some caveats (Robert et al. 2011). Here, type I error represents false negative rate – i.e. the number of PODs modelled under a given scenario that are misclassified; type II error represents false positive rates – i.e. the number of PODs produced under a different scenario that are mistaken for the true scenario.

Figure A2 shows the mean true rate at the hierarchical selection step for each divergence scenario under all combinations of summary statistics and methods. Clearly the neural network and multinomial logistic methods outperform basic rejection at all tolerances and all summary statistics. With 20 summary statistics and 1% tolerance, mean true rate (± SD) for all scenarios was 0.83±0.06 for neural network rejection versus 0.75±0.06 for multinomial logistic regression (Fig A2). Furthermore, 20 summary statistics and 1% tolerance showed low Type I and Type II error for both these methods while basic rejection remained extremely unreliable (Figs A3 & A4). Although using 29 summary statistics marginally improved true rate for neural network rejection (0.84±0.06), PCA indicated that the increased dimensionality meant that simulated datasets no longer properly reflected the observed data (Fig A5). Therefore hierarchical model selection within divergence scenarios was performed using 20 summary statistics, neural network rejection and 1% tolerance. Posterior probabilities for different growth models are shown in Table A4.

Final model selection

POD analysis of final model selection showed a similar pattern to the hierarchical analyses – i.e. a marked improvement of both the multinomial logistic regression and neural network methods over standard rejection

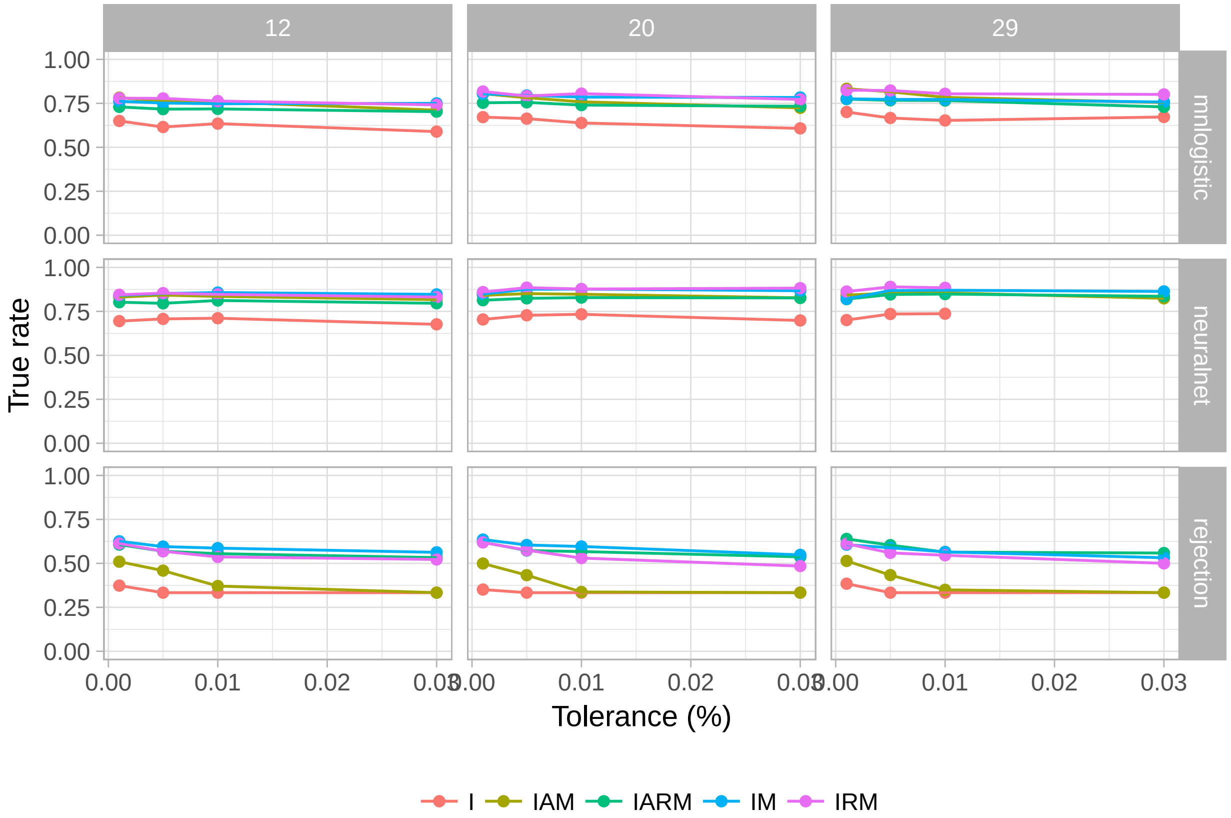

Figure A2: Mean probability (per divergence scenario) of true model being chosen under different summary statistic and rejection method comparisons.

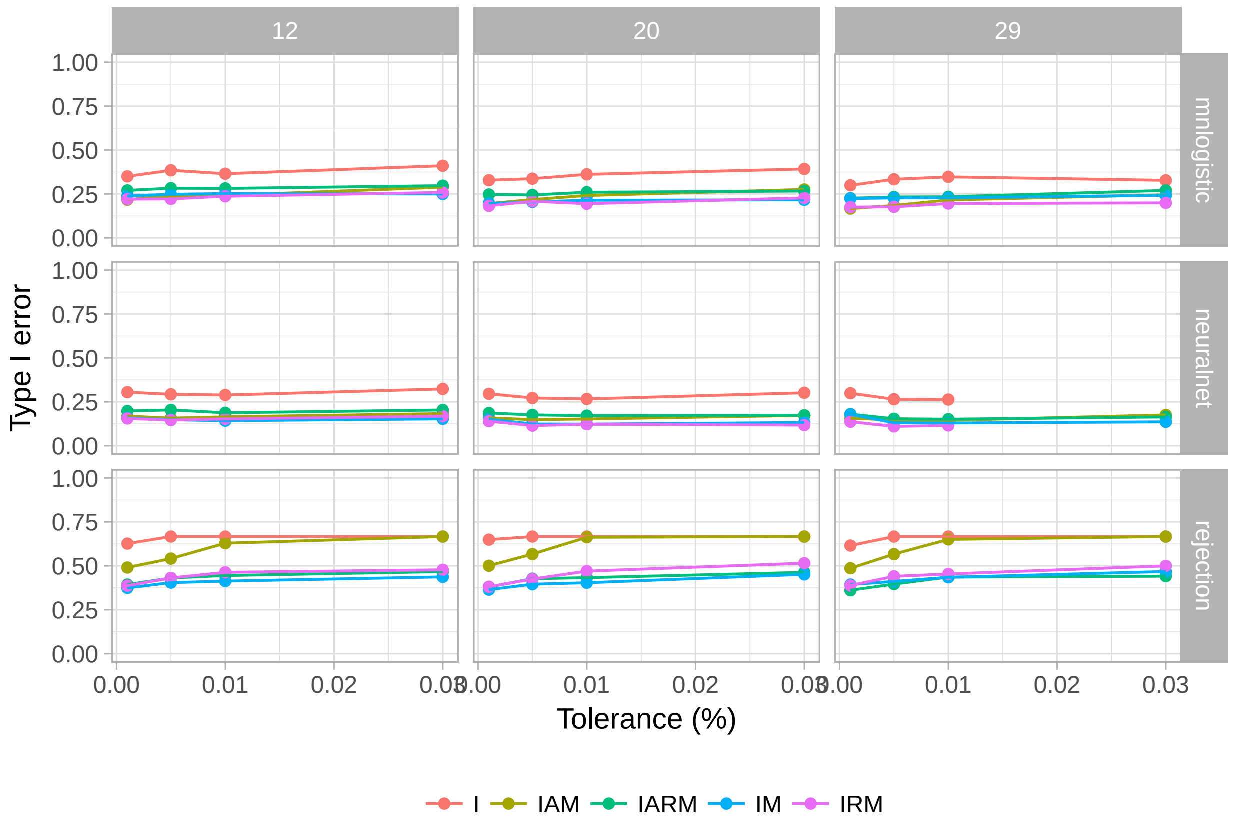

Figure A3: Mean Type I error rate for each divergence scenario under different summary statistic and rejection method comparisons. Here Type I error is the proportion of PODs simulated under a scenario mistaken for an alternative scenario.

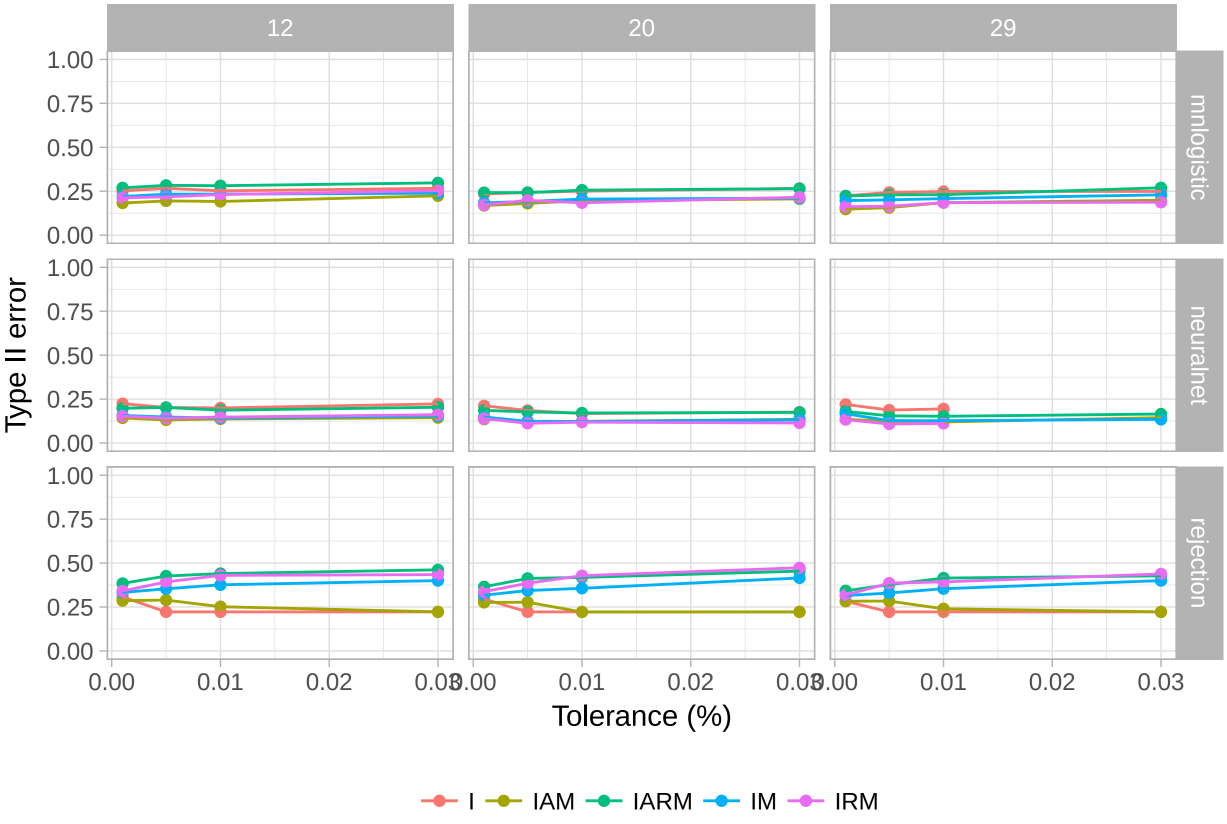

Figure A4: Mean Type II error rate for each divergence scenario under different summary statistic and rejection method comparisons. Here Type II error is the proportion of PODs simulated under a different scenario mistaken for the true scenario.

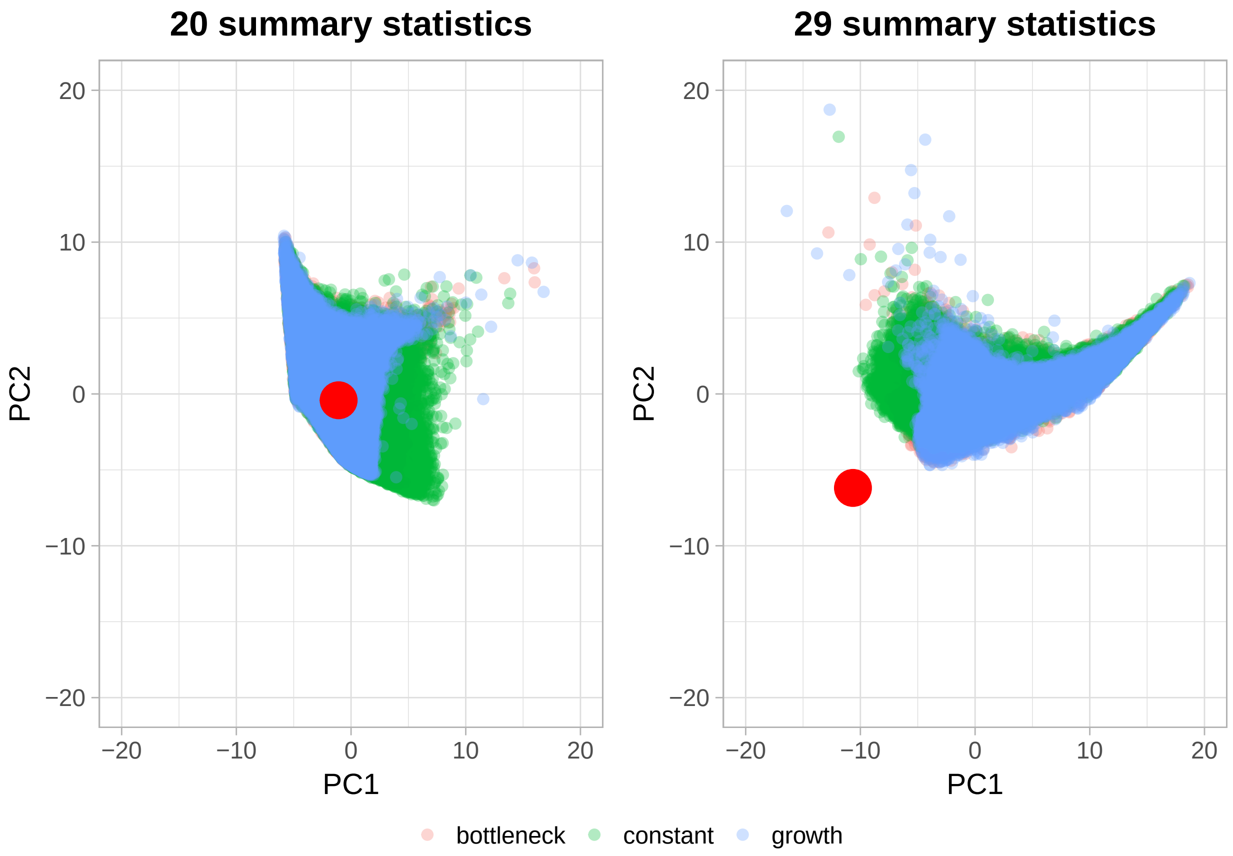

Figure A5: Principal component analysis of summary statistics from observed and simulated datasets for the IM model; observed dataset denoted by large red point. (NB. PCA was performed separately in both cases, hence the reason for the different position of the observed dataset in the two panels).

Model	Bottleneck	Constant	Growth
I	0.286	0.444	0.270
IM	0.867	0.008	0.125
IAM	0.161	0.599	0.240
IARM	0.643	0.003	0.354
IRM	0.556	0.000	0.444

Table A4: Posterior probability estimates from hierarchical model selection within divergence scenarios with 20 summary statistics, 1% tolerance and neural network rejection.

(Fig A6). Of the two, neural network rejection was superior with higher probability of the true model being chosen - although the power of the analysis varied with divergence scenarios (Fig A6). Selection using 20 summary statistics, instead of 12 performed slightly more efficiently.

Although identifying the correct divergence scenario is a key aim of our study, we are primarily interested in determining whether divergence has occurred with or without gene flow. Therefore we calculated the probability of our ABC framework identifying a scenario of pure isolation when migration has in fact occurred. The probability of this was generally low across all rejection methods, tolerance rates and summary statistic combinations, but was lowest for the neural network method (Fig A7).

Surprisingly however the probability for the opposite scenario, i.e. falsely detecting gene flow when none has occurred, was high irrespective of the method, summary statistic or tolerance rate used (red lines, Fig A7). Closer inspection of confusion matrices among models indicated that the reason for this is that our ABC approach has difficulty distinguishing the isolation and isolation with ancient migration models (Fig A8 shows the frequency of confusion for different rejection methods). In other words, these two models produce very similar summary statistics.

To account for this, we recalculated false detection probabilities with the IAM model removed (blue lines, Fig A7). This clearly shows that the neural network method minimises both false detection probabilities across all tolerance rates - however the rejection method still has trouble distinguishing isolation and migration scenarios, even at low tolerance rates.

We conducted final model selection using the five models chosen during hierarchical selection, the neural network rejection method and 20 summary statistics. Posterior probability estimates overwhelmingly supported a model of isolation with migration with a bottleneck in the Japan Sea across all tolerance values (see Table 2 in the main text). Comparing the distribution of the summary statistics from the accepted datasets with our observed data confirmed our framework produced realistic estimates of these statistics (Fig A9).

Parameter estimation accuracy

We additionally used PODs approach to assess how well we were able to estimate parameters under the chosen model using the neural network and standard rejection methods. Using the R *abc* `cv4abc` function, we produced 100 PODs for the IM model using two different tolerance levels (1 and 3% of the simulated data) and both 12 and 20 summary statistic subsets. This allowed us to compare estimated vs. the true parameter values used to perform the simulations. For the theta and divergence time parameters shown in Figure A10, the number of summary statistics did not appear to influence parameter estimates. However, increasing tolerance did increase the deviation of estimated parameters from their true valus. Conservatively, we chose to use the neural network, 20 summary statistics and 1% tolerance.

Examining introgression in more detail

Overlap between introgression and loci used in ABC

Our study finds evidence of introgression at small, localised regions throughout the genome. If these introgressed regions are overrepresented in the observed loci used for our ABC analysis, they may bias our model

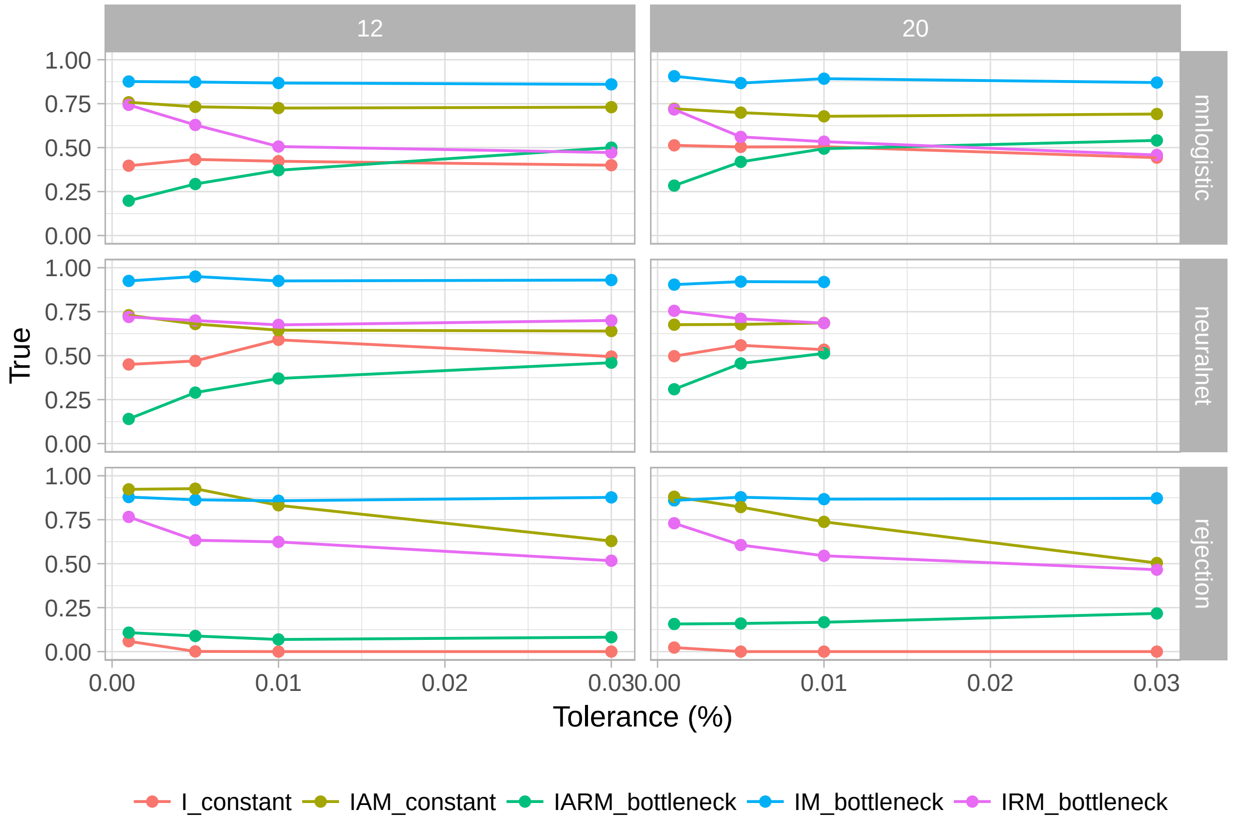

Figure A6: Mean probability (per divergence scenario) of true model being chosen under different summary statistic and rejection method comparisons for the final model selection step

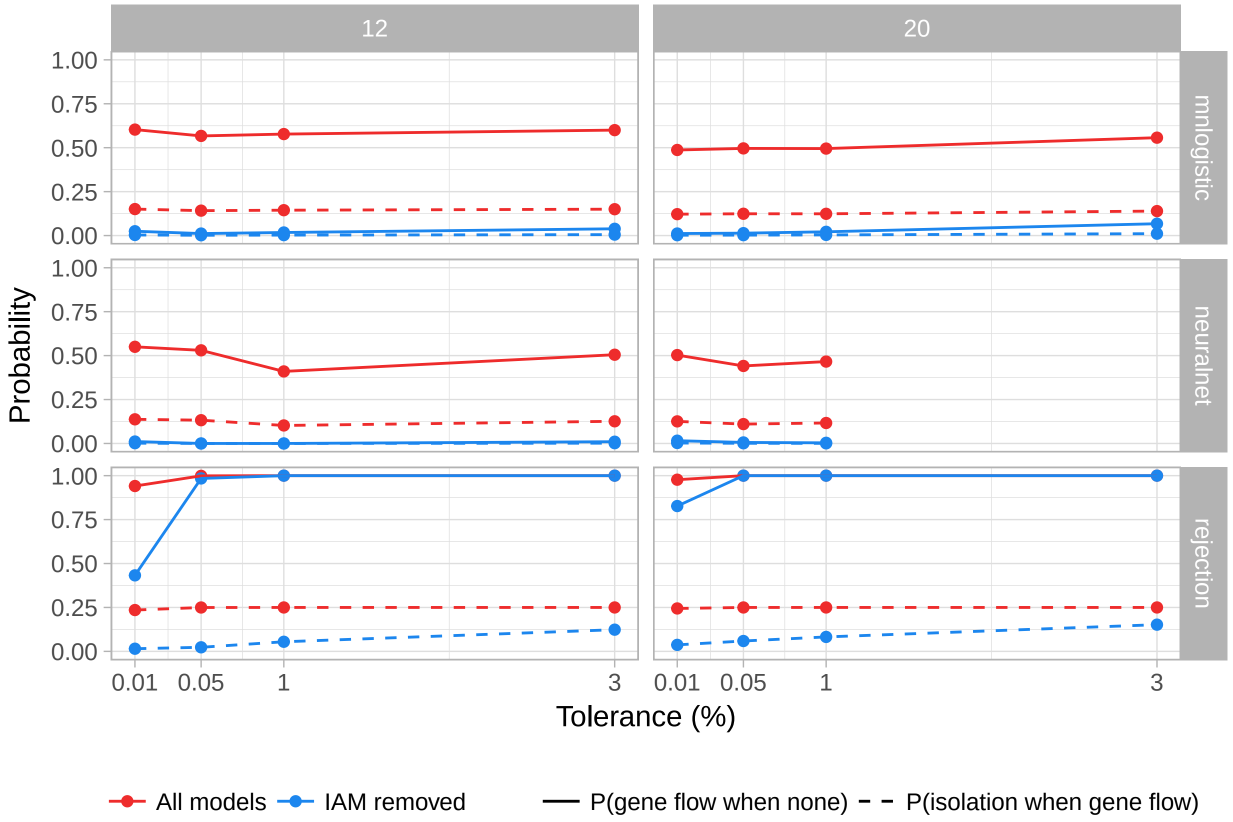

Figure A7: Probability of falsely detecting gene flow or no gene flow for different combinations of summary statistics, rejection methods and tolerance values.

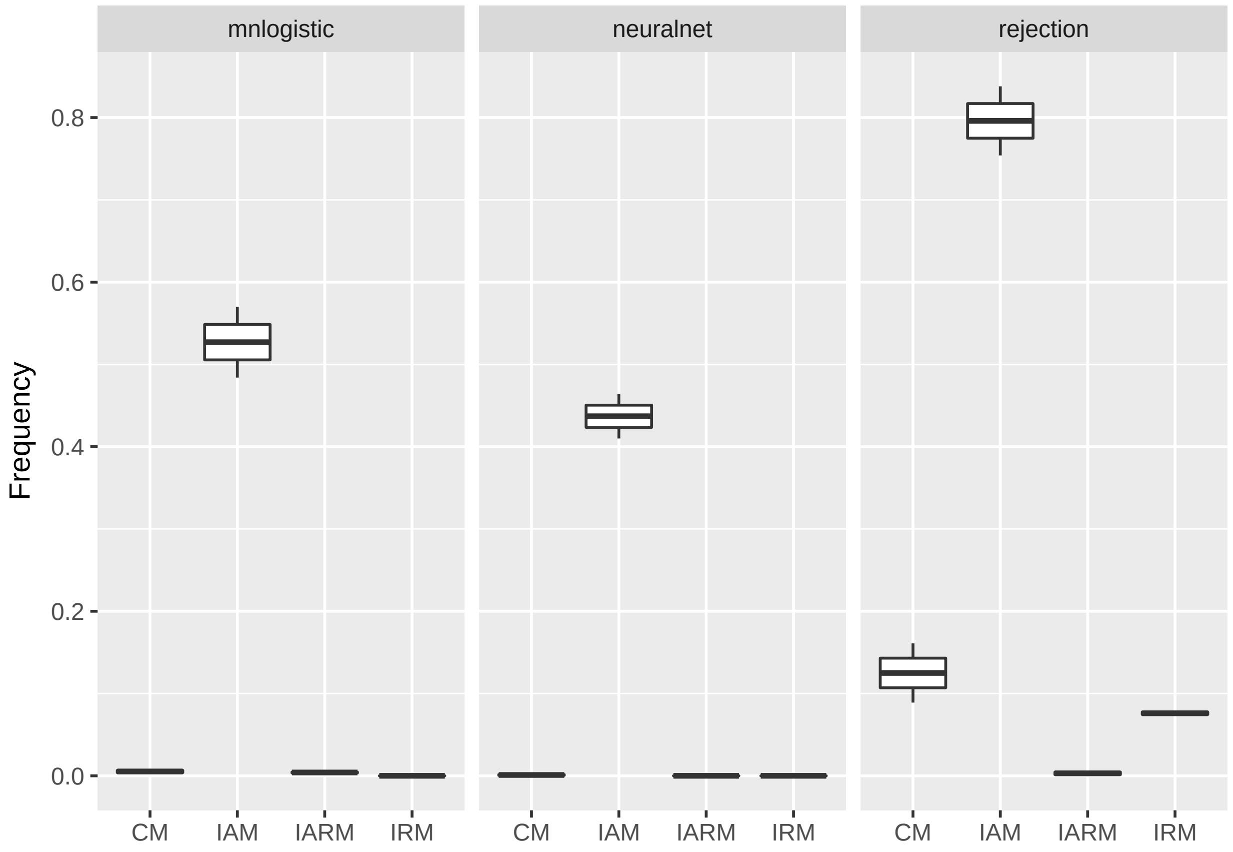

Figure A8: Frequency of confusion between isolation and all other models across methods and tolerances

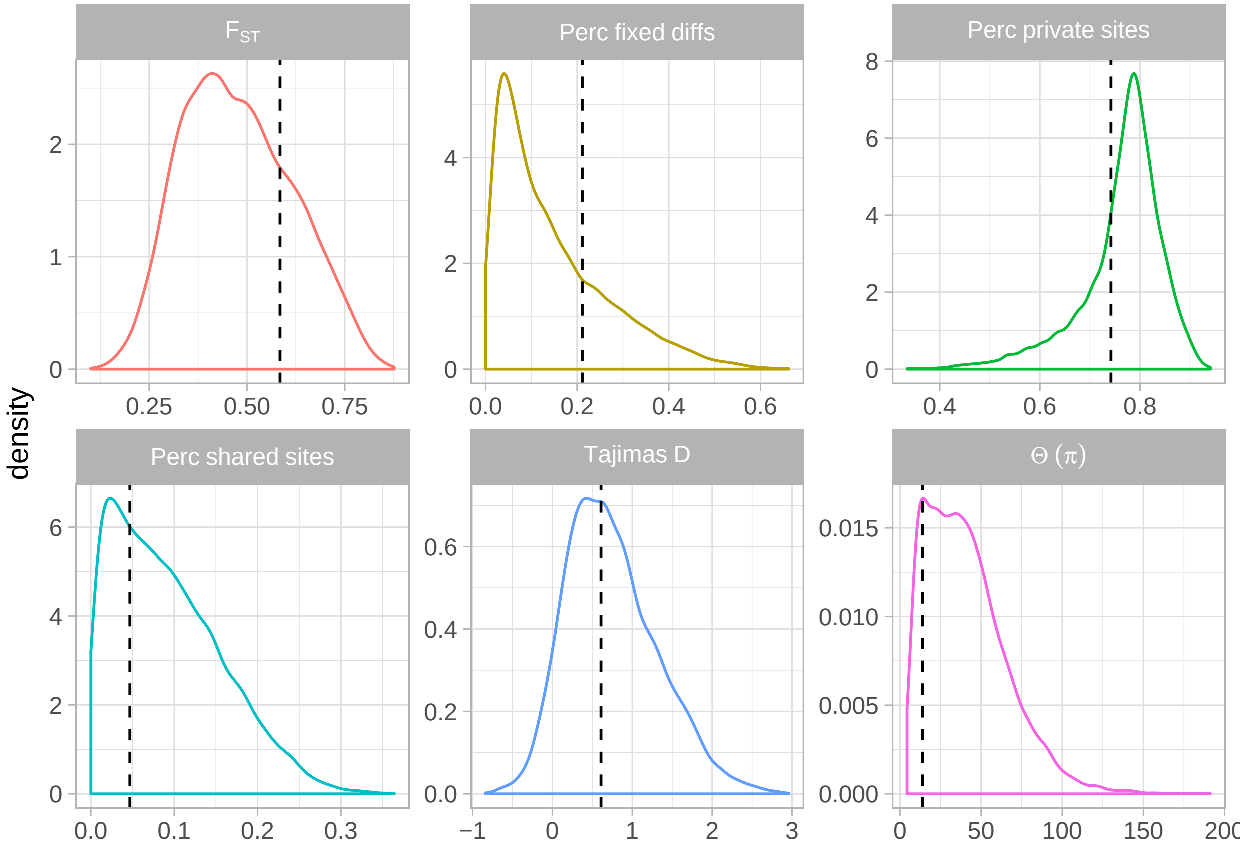

Figure A9: Distributions of accepted summary statistics from the simulation step of the final chosen model (IM). Values of observed summary statistics are indicated by the dashed vertical lines.

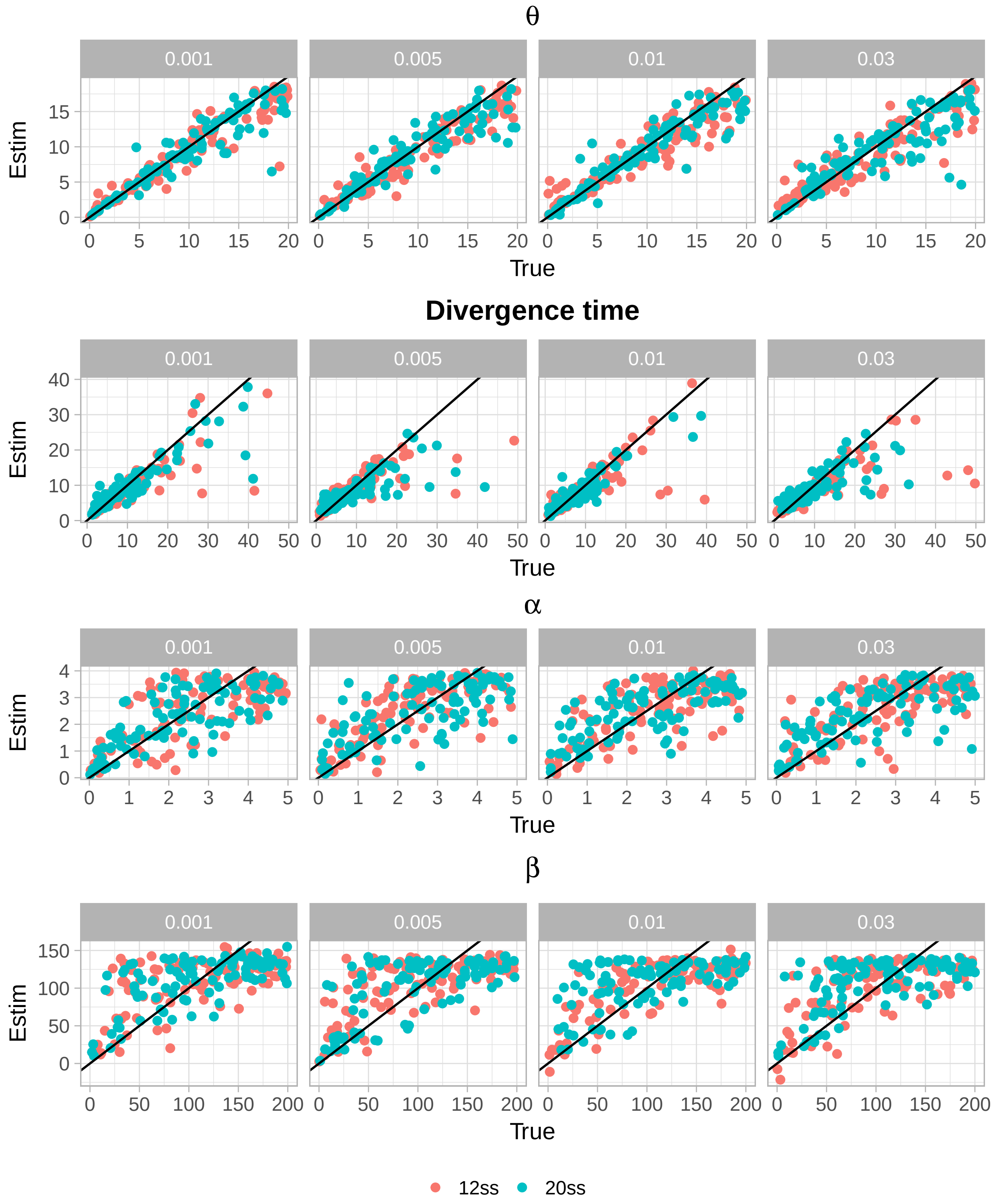

Figure A10: True vs estimated parameter values for theta, divergence time and migration rate hyperprior parameters based on 100 PODs.

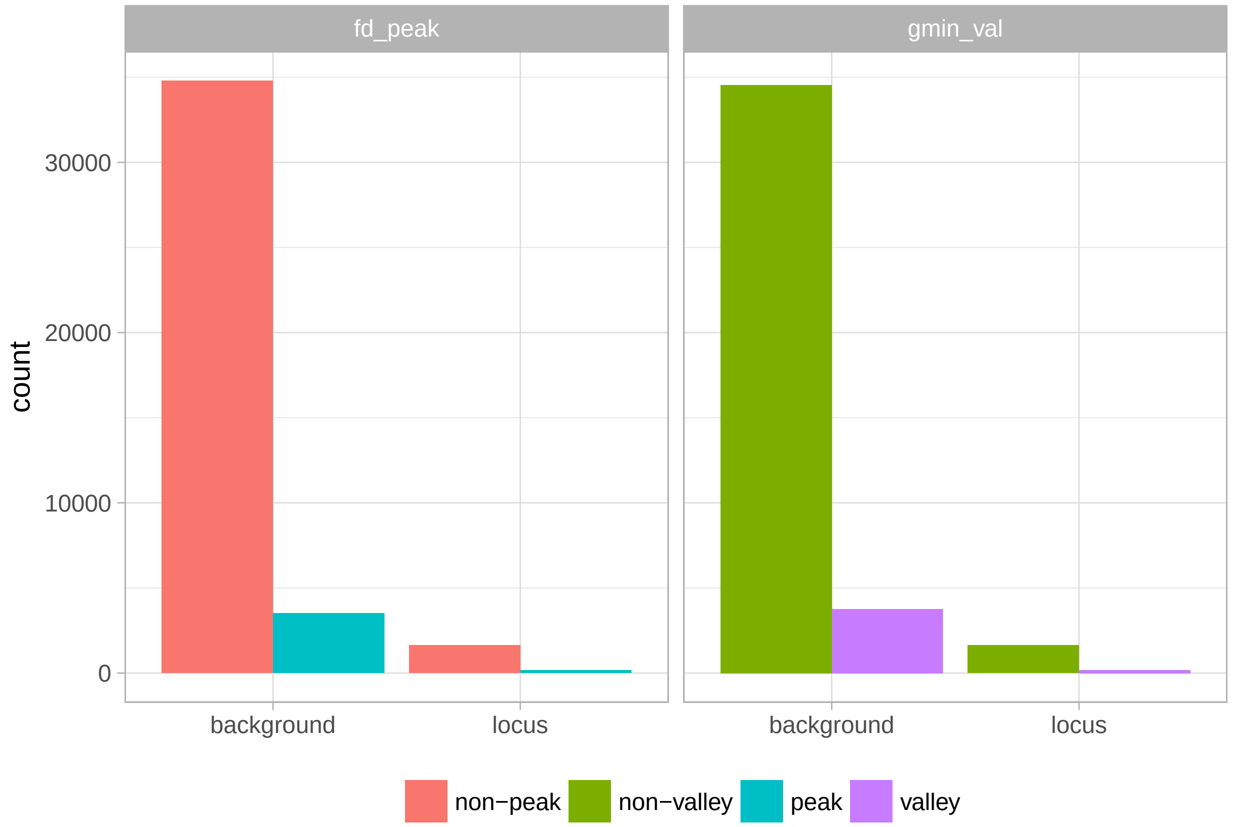

Figure A11: Barplot of proportions of f_{d} peaks or G_{MIN} valleys among ABC loci vs the genome background

selection towards those with gene flow. In order to assess whether this is the case, we examined whether the 1874 ABC loci fell in a higher proportion of introgressed regions than expected, given that they are a random sample from the genome.

As Figure A11 shows, the proportion of ABC loci falling in either f_{d} peaks or G_{MIN} valleys is equivalent to the genome background. For both types of introgression region, 95% of the genome is background - i.e. showing no evidence of increased gene flow; this proportion is identical in the ABC loci (proportional tests, G_{MIN}: $\chi = 0.037$, df = 1, $P = 0.845$; f_{d}: $\chi = 0.165$, df = 1, $P = 0.684$).

Figure A12 also clearly shows no difference between the distribution of G_{MIN} estimated from the genome and from the windows only containing ABC loci. This suggests our observed dataset does not introduce bias into the analysis.

Posterior distribution of migration hyperpriors

In addition to the posterior estimates of migration rates given in the main text, we provide further detail here on the posterior distributions of the migration hyperpriors. These were used to ensure that genome-wide migration is heterogeneous (Roux et al. 2013; Roux et al. 2014). Fig A13 shows the posterior distributions for the β distribution parameters and the unscaled distribution itself. Note that the two individual migration rate parameters m_{12} and m_{21} (i.e. JS>PO and PO>JS - see Table A1) are scaled by a scalar c, drawn independently for both rates - see Figure 2 in the main text for the scaled posterior β distributions.

From this figure, we can see that under our IM model, a large number of loci experience a small amount of gene flow.

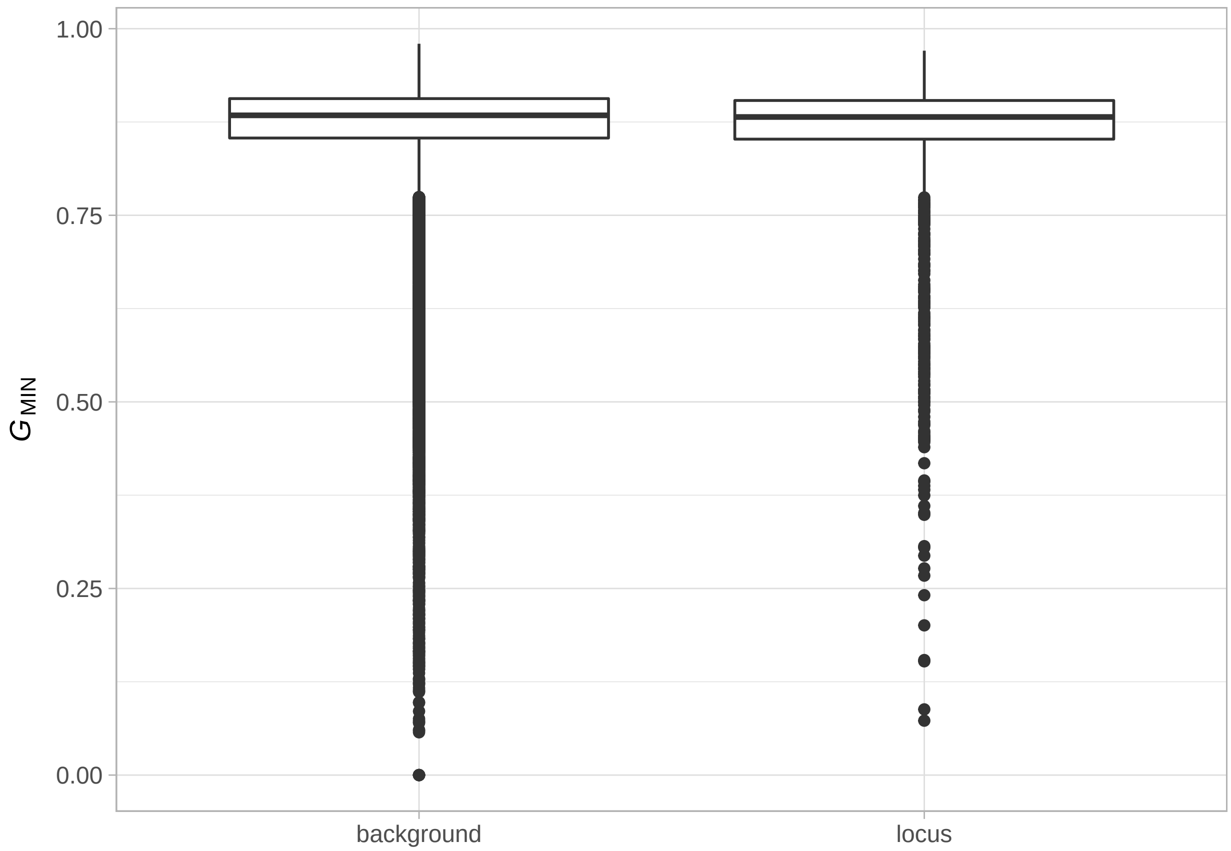

Figure A12: Comparison of G_{MIN} between ABC loci and the genome background

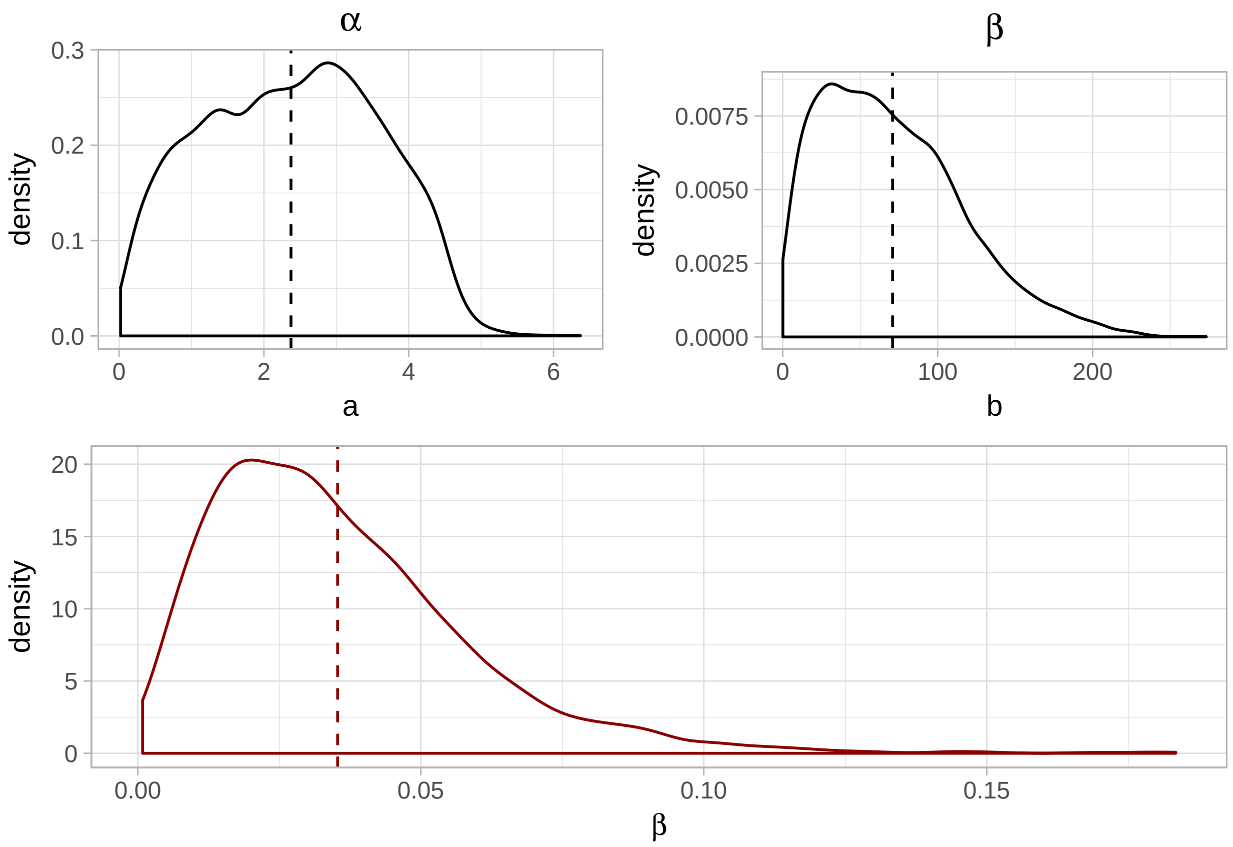

Figure A13: Examination of migration hyperpriors and the unscaled beta distribution; upper panels are posterior distributions for α and β, the hyperprior distribution parameters used to estimate the unscaled beta distribution; lower panel is unscaled β distribution, based on 1800 samples of a β distribution with the posterior estimates of α and β. Individually scaled distributions for JS>PO and PO>JS can be seen in Figure 2 of the main text. Dashed vertical lines denote distribution means.

Demographic estimation using the site-frequency spectrum

In addition to our ABC analysis of demographic inference, we used a maximum likelihood method approach based on the site-frequency spectrum implemented in fastsimcoal2 (Excoffier et al. 2013). For this, we used RAD-seq data from the Japan Sea and Pacific Ocean individuals ($n = 51$). To account for missing data, we resampled 20 genotypes per species at each site, resulting in calls for all 20 'pseudo-individuals' at 22,065 SNP loci. We converted genotype calls to a folded joint site-frequency spectrum using arlequin.

	Description	Prior	Models
N_{PO}	JS effective population size	loguniform; 10,000 - 1,000,000	All
N_{JS}	PO effective population size	loguniform; 10,000 - 1,000,000	All
T_{DIV}	Divergence time	loguniform; 10,000 - 2,000,000 years	All
T_{am}	Timing of end of ancient migration (proportion)	loguniform; 0.01-0.3	IAM, IARM
T_{rm}	Timing of onset of recent migration (proportion)	loguniform; 0.7-1	IRM, IARM
m_{12}	Migration rate JS>PO	loguniform; 1 x 10^{-1} - 0.01	All except isolation
m_{21}	Migration rate PO>JS	loguniform; 1 x 10^{-1} - 0.01	All except isolation

Table A5: Parameters and parameter search ranges used in divergence scenarios for maximum likelihood SFS estimation.

We used the same models as used for ABC (see Fig A1); however in order to simplify this analysis, we used only two models of growth - constant population size and a bottleneck in the Japan Sea. Since maximum-likelihood analysis does not make use of priors (in comparison to the Bayesian approach in our ABC analysis), we instead provided loguniform parameter search ranges for each model to fastsimcoal2 (see Table A5) and assumed a SNP mutation rate of 7.1 x 10^{-9} per site per year (Guo et al. 2013). For each model we performed 100 independent runs of 100,000 coalescent simulations. Model selection was carried out on the run with the highest likelihood using AIC. However, following Meier et al. (2017), we also calculated the likelihood distribution for each model using 100 expected site frequency spectra and 1,000,000 coalescent simulations.

Model	Growth	logLikelihood	N parameters	AIC	deltaAIC	deltaLL
IARM	bottleneck	-18032.835	15	36095.670	0.000	1418.999
IRM	bottleneck	-18360.822	12	36745.644	649.974	1746.986
isolation_migration	bottleneck	-18703.335	10	37426.670	1331.000	2089.499
IAM	bottleneck	-19019.648	12	38063.296	1967.626	2405.812
isolation	bottleneck	-19021.768	8	38059.536	1963.866	2407.932
IRM	constant	-18700.429	9	37418.858	1323.188	2086.593
IARM	constant	-18704.933	12	37433.866	1338.196	2091.097
IAM	constant	-19010.365	9	38038.730	1943.060	2396.529
isolation	constant	-19013.523	5	38037.046	1941.376	2399.687
isolation_migration	constant	-19018.089	7	38050.178	1954.508	2404.253

Table A6: Model selection using AIC and logLikelihood.

Model selection using AIC and log likelihood values showed strongest support for an IARM model with a population expansion in the Japan Sea lineage following a bottleneck (Table A6). It should be noted that since we did not use independent polymorphic sites to generate our SFS, AIC results must be interpreted with caution (Excoffier et al. 2013). However, likelihood distributions for each of the models confirm that the IARM bottleneck model is best supported by the data. Furthermore, inclusion of a more realistic population growth model clearly improved our ability to distinguish between demographic scenarios (Figure A14). Parameter estimates from the IARM bottleneck model are shown in Table A7. Our SFS analysis suggests an ancient divergence of ~1.2 million years for the Japan Sea and Pacific, older than our ABC analyses. Ancient migration ceased 3 Kyr after divergence and then recent migration began around 13 kyr BP concordant with contact after the LGM, whereas a bottleneck occured 0.66 million years ago (Table A7). As with our ABC analysis, the SFS based inference suggests the migration rate from the Japan Sea into the Pacific Ocean (m_{12}) is lower than migration from the Pacific Ocean into the Japan Sea (m_{21}; Table A7).

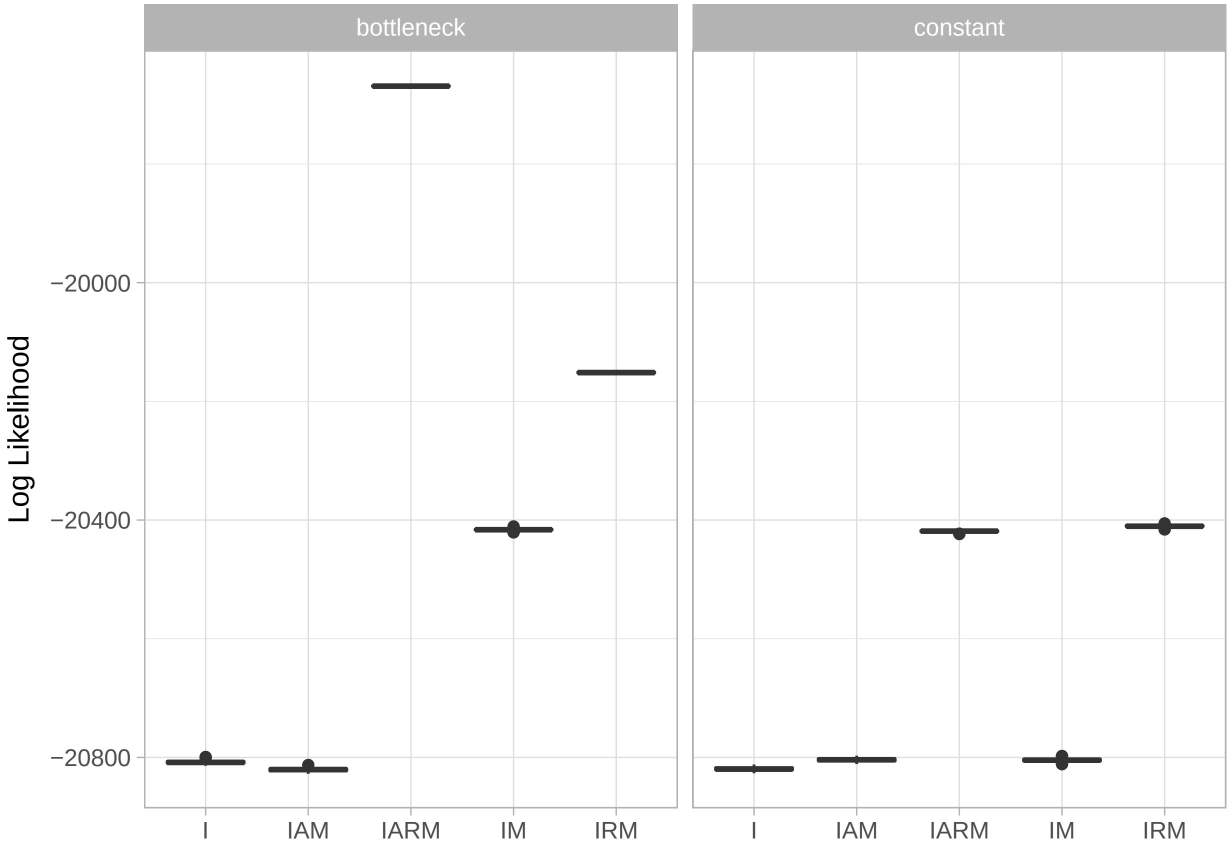

Figure A14: Log-likelihood distribution overlap based on expected SFS

Some discrepancy between our ABC analyses and SFS estimation is expected given the different nature of the methods, the priors/parameter search ranges used and the use of datasets derived from different sources (whole genome-resequencing vs RAD-seq). One major difference is that our SFS analysis assumes a mutation rate of 7.1 x 10^{-9} per site per year whereas our ABC analysis does not estimate mutation rate and instead scales parameter estimates to an arbitary value of N_e - set at 50,000. An additional difference between the two approaches is that for ABC, we assumed heterogeneous migration rates - i.e. we allowed migration rate to vary among loci, as might be expected in a situation of speciation-with-gene-flow (Roux et al. 2013; Roux et al. 2014). In contrast, our SFS approach assumed only a single, uniform migration rate across the genome. Not incorporating migration rate heterogeneity can substantially alter parameter estimates between otherwise equivalent models (Roux et al. 2013; Roux et al. 2014) and this likely explains why migration rate is greater in the SFS analysis. Importantly however, divergence time etimates from the SFS analysis are within the 95% CI of divergence times from our ABC analysis (0.18-4.1 million years); this is also true for the timing of the bottleneck (0.03 - 2 million years).

Model	N_{PO}	N_{JS}	T_{DIV}	T_{am}	T_{rm}	T_{BOT}	N_{BOT}	m_{12}	m_{21}
bottleneck	1137659	1147457	1216225	1213496	13103	660304	56270	4.21×10^{-5}	4.84×10^{-5}

Table A7: Parameters and priors used in divergence scenarios for maximum likelihood SFS estimation.

Effect of phasing and altering missing data thresholds

An important point for our introgression analysis is to ensure that missing base calls or insufficient filtering cannot account for the observed patterns (using G_{MIN} & f_{d}) we see in our data. For both phased and unphased datasets (see main text for more details on these datasets), we performed G_{MIN} & f_{d} analyses using a minimum of 5000 sites per 10 Kb window. The average number of useable sites (i.e. passing all quality filtering and depth thresholds) was 6677 for the phased dataset and 8732 for the unphased data. These differences can be accounted for by the fact that more stringent filtering is necessary for statistical phasing to be accurate.

From the joint distribution of our introgression statistics with the number of sites per analysed window (see Fig A15), there is no clear relationship in either phased or unphased datasets. Therefore it seems unlikely that missing data can explain the presence of G_{MIN} valleys or f_{d} peaks in our ananlysis.

We also examined how both statistics varied along an example chromosome (chrIV) using phased and unphased datasets and also with different filtering threshold. For G_{MIN}, it is clear that unphased data has a tendency to produce more windows with a lower value of the statistic but remains robust to increasing missing data filters (Fig A16). In contrast, phased data appears to be somewhat more conservative, most likely because of the need to apply more stringent filters (Fig A16). Nonetheless, phased data clearly identifies similar extreme G_{MIN} valleys - irrespective of increased data filtering. Therefore, it is reasonable to conclude that the recent introgression we detect with our G_{MIN} approach is not an artefact of dating handling.

We also examined the robustness of f_{d}, a different measure of introgression on chrIV to phasing and filtering. There are few quantative differences between phased and unphased data for f_{d}, with peaks clear in both datasets. In fact, phasing data appears to increase the extent of f_{d} peaks (Fig A17), suggesting our unphased approach may be more conservative.

References

Blum, Michael G. B., and Olivier François. 2010. "Non-linear regression models for Approximate Bayesian Computation." *Statistics and Computing* 20 (1): 63–73. doi:10.1007/s11222-009-9116-0.

Excoffier, Laurent, Isabelle Dupanloup, Emilia Huerta-Sánchez, Vitor C Sousa, and Matthieu Foll. 2013. "Robust demographic inference from genomic and SNP data." *PLoS Genetics* 9 (10): e1003905.

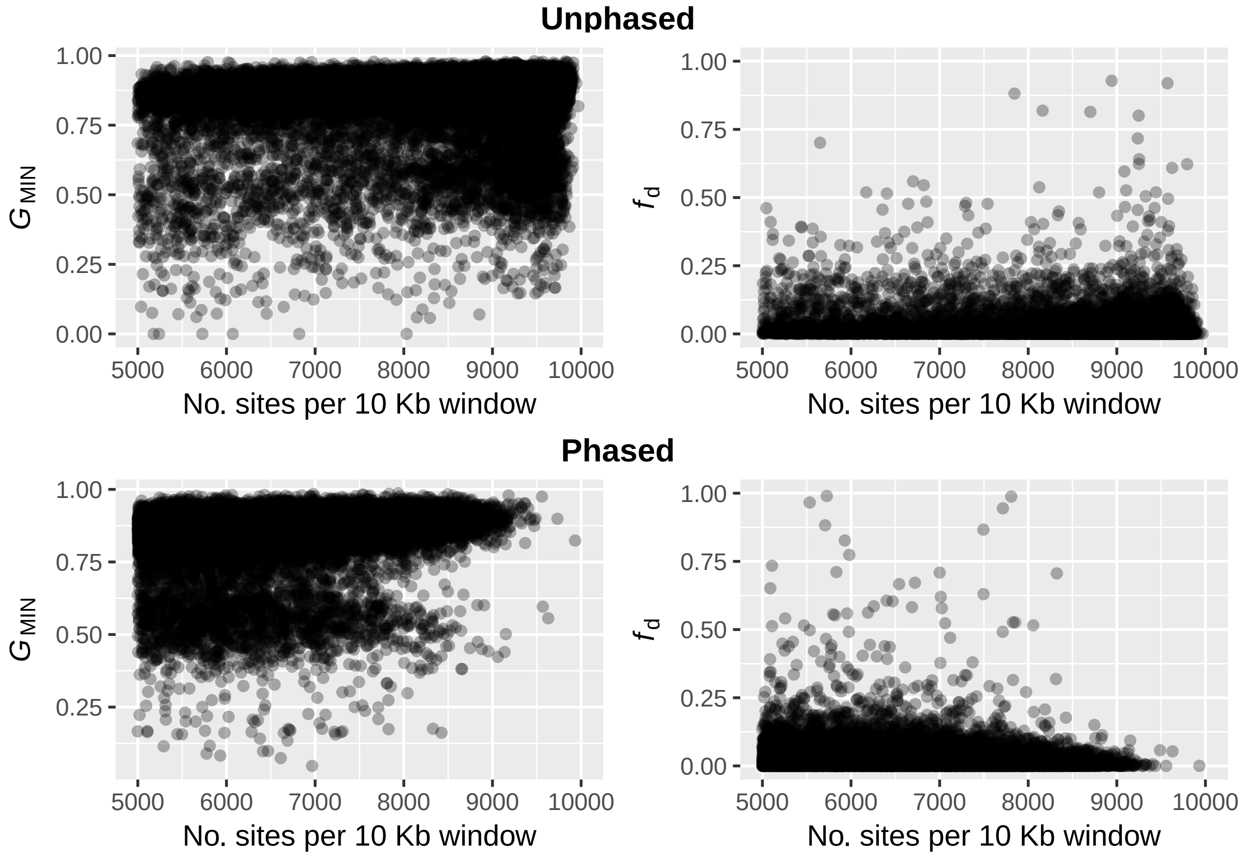

Figure A15: Joint distribtuion of introgression statistics with minimum number of sites allowed per 10 Kb genome window

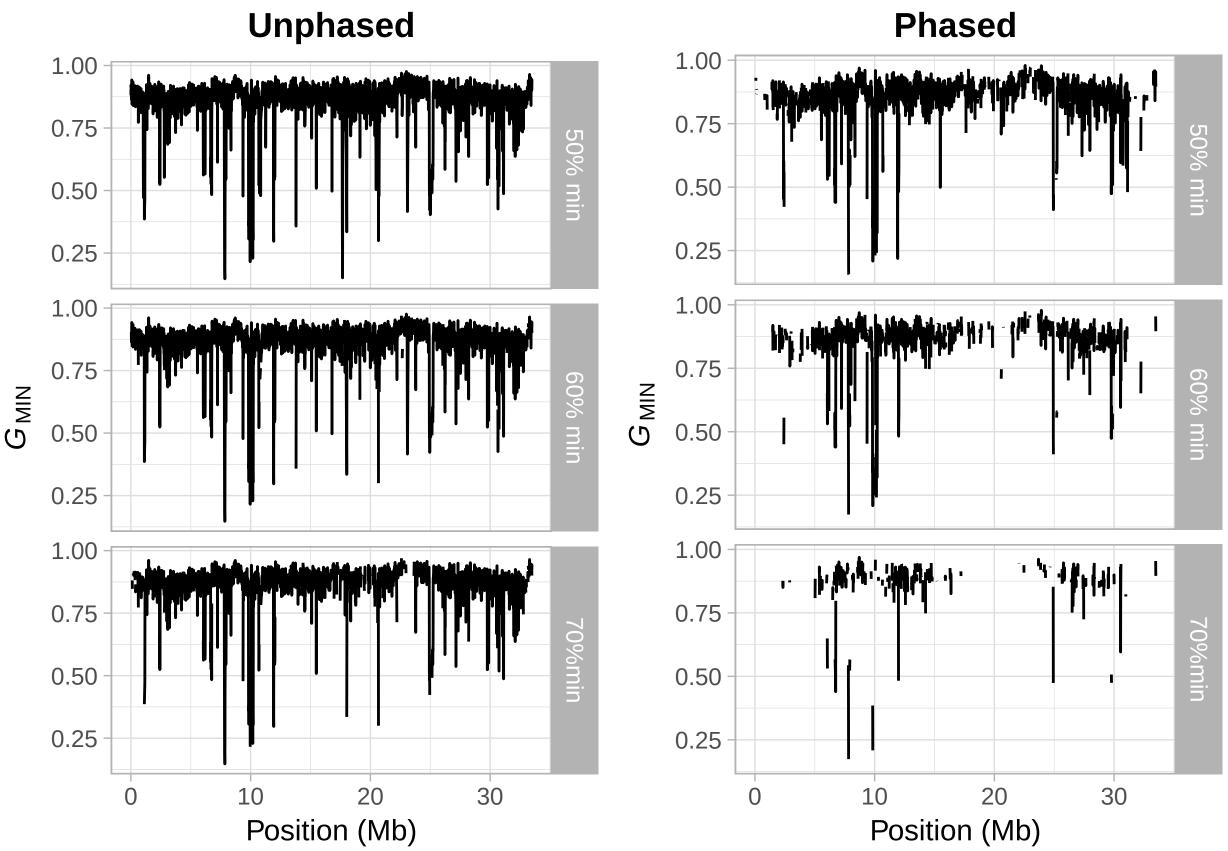

Figure A16: Variation in 10 Kb estimates of G_{MIN} across chrIV with different minimum data thresholds and a comparison between phased and unphased data

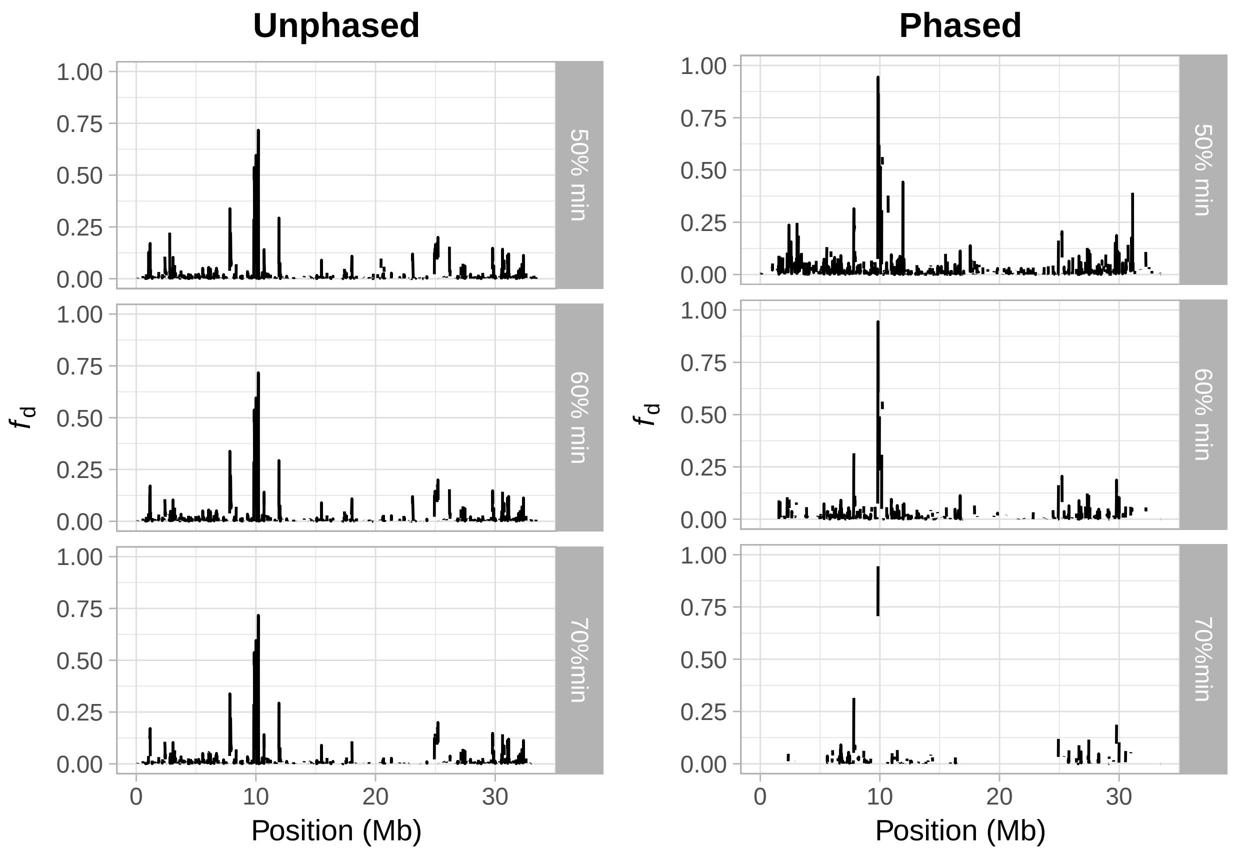

Figure A17: Variation in f_d with different minimum data thresholds and a comparison between phased and unphased data

doi:10.1371/journal.pgen.1003905.

Fagundes, Nelson J R, Nicolas Ray, Mark A Beaumont, Samuel Neuenschwander, Francisco M Salzano, Sandro L Bonatto, and Laurent Excoffier. 2007. "Statistical evaluation of alternative models of human evolution." *Proceedings of the National Academy of Sciences of the United States of America* 104 (45): 17614–9. doi:10.1073/pnas.0708280104.

Guo, Baocheng, Frédéric JJ Chain, Erich Bornberg-Bauer, Erica H. Leder, and Juha Merilä. 2013. "Genomic Divergence Between Nine- and Three-Spined Sticklebacks." *BMC Genomics* 14 (1): 756. doi:10.1186/1471-2164-14-756.

Meier, Joana I., Vitor C. Sousa, David A. Marques, Oliver M. Selz, Catherine E. Wagner, Laurent Excoffier, and Ole Seehausen. 2017. "Demographic modelling with whole-genome data reveals parallel origin of similar Pundamilia cichlid species after hybridization." *Molecular Ecology* 26 (1): 123–41. doi:10.1111/mec.13838.

Nadachowska-Brzyska, Krystyna, Reto Burri, Pall I. Olason, Takeshi Kawakami, Linnéa Smeds, and Hans Ellegren. 2013. "Demographic Divergence History of Pied Flycatcher and Collared Flycatcher Inferred from Whole-Genome Re-sequencing Data." Edited by Bret A. Payseur. *PLoS Genetics* 9 (11): e1003942. doi:10.1371/journal.pgen.1003942.

Nielsen, Rasmus, and Mark a Beaumont. 2009. "Statistical inferences in phylogeography." *Molecular Ecology* 18 (6): 1034–47. doi:10.1111/j.1365-294X.2008.04059.x.

Pavlidis, P, S Laurent, and W Stephan. 2010. "msABC: a modification of Hudson's ms to facilitate multi-locus ABC analysis." *Molecular Ecology Resources* 10 (4): 723–7. doi:10.1111/j.1755-0998.2010.02832.x.

Robert, C. P., J.-M. Cornuet, J.-M. Marin, and N. S. Pillai. 2011. "Lack of confidence in approximate Bayesian computation model choice." *Proceedings of the National Academy of Sciences* 108 (37). doi:10.1073/pnas.1102900108.

Robinson, John D., Lynsey Bunnefeld, Jack Hearn, Graham N. Stone, and Michael J. Hickerson. 2014. "ABC inference of multi-population divergence with admixture from un-phased population genomic data." *Molecular Ecology* 23 (August): 4458–71. doi:10.1111/mec.12881.

Roux, C., C. Fraïsse, V. Castric, X. Vekemans, G. H. Pogson, and N. Bierne. 2014. "Can we continue to neglect genomic variation in introgression rates when inferring the history of speciation? A case study in a Mytilus hybrid zone." *Journal of Evolutionary Biology* 27 (8): 1662–75. doi:10.1111/jeb.12425.

Roux, C., Georgia Tsagkogeorga, Nicolas Bierne, and Nicolas Galtier. 2013. "Crossing the species barrier: genomic hotspots of introgression between two highly divergent Ciona intestinalis species." *Molecular Biology and Evolution* 30 (7): 1574–87. doi:10.1093/molbev/mst066.

Tange, O. 2011. "GNU Parallel - the Command-Line Power Tool."*;Login: The USENIX Magazine* 36 (1). Frederiksberg, Denmark: 42–47. http://www.gnu.org/s/parallel.
